# Mental health crisis situations: the nurse’s work in Primary Health Care ^*^

**DOI:** 10.1590/1518-8345.7015.4356

**Published:** 2024-10-25

**Authors:** Gessner Bravo de Paula, Nur Mohamad Ali El Akra, Lucas Ferraz Córdova, Lucilene Cardoso, Ana Carolina Guidorizzi Zanetti, Bianca Cristina Ciccone Giacon Arruda

**Affiliations:** ^1^ Universidade Federal do Mato Grosso do Sul, Instituto Integrado de Saúde, Campo Grande, MS, Brazil.; ^2^ Secretaria do Estado de Saúde, Coordenadoria Estadual do Telessaúde, Campo Grande, MS, Brazil.; ^3^ Scholarship holder at the Fundação de Apoio ao Desenvolvimento do Ensino, Ciência e Tecnologia do Estado do Mato Grosso do Sul (FUNDECT), Brazil.; ^4^ Scholarship holder at the Coordenação de Aperfeiçoamento de Pessoal de Nível Superior (CAPES), Brazil.; ^5^ Universidade Federal do Mato Grosso do Sul, Faculdade de Ciências Humanas, Campo Grande, MS, Brazil.; ^6^ Universidade de São Paulo, Escola de Enfermagem de Ribeirão Preto, PAHO/WHO Collaborating Centre for Nursing Research Development, Ribeirão Preto, SP, Brazil.

**Keywords:** Primary Health Care, Nurses, Crisis Intervention, Healthcare Models, Mental Health, Work

## Abstract

**Objective:**

to analyze the nurses’ work in Primary Health Care in the face of mental health crisis situations.

**Method:**

this is a descriptive-exploratory study with a qualitative approach, supported by the theoretical-interpretive frameworks of behavior analysis and historical-dialectical materialism. The data was collected through a semi-structured interview with twelve Primary Health Care nurses and analyzed using the deductive technique proposed by the Theorical Domains Framework, the methodological reference adopted.

**Results:**

the data analyzed made it possible to draw up two themes: “Nurses’ work: material and social determinants” and “Nurses’ subjective conditions in the face of mental health crisis situations”. Nurses’ work was guided by the protocol execution of “technical” steps related to clinical psychiatry, with an understanding of the crisis as a “psychiatric outbreak”.

**Conclusion:**

the study made it possible to analyze the nurse’s work in crisis situations, describing the objective and subjective contradictions, their understanding of the crisis phenomenon and the emotional repercussions of this work on the professionals. This situation raises the need for action, organization and political-social mobilization of the nursing category in the fight against the asylum model of care and the consolidation of the Psychiatric Reform perspective.

## Highlights

**(1)** Manicomial protocol action to identify and reduce crisis symptoms.

**(2)** Reducing the experience of crisis as a psychopathological phenomenon.

**(3)** Use of theoretical-critical references aligned with the proposals of psychosocial care.

**(4)** Internationally validated methodological and analytical framework.

**(5)** The need for political and social action on the nursing profession’s part.

## Introduction

 Mental health impairment has shown significant growth worldwide among the general population. In 2019, it was estimated that approximately 13% of the world’s population (970 million people) lived with some form of mental health impairment, the most prevalent being anxiety disorders (31%), depression (28.9%) and those resulting from the use of alcohol and other drugs ^(^ ^1^ ^)^ . 

 Mental health is defined by the World Health Organization as a state in which the individual is able to function, thrive, cope with difficulties, connect and contribute to the improvement of their community. Therefore, these problems are closely related to/determined by physical, biological, sociocultural and behavioral conditions ^(^ ^2^ ^-^ ^4^ ^)^ . 

 In addition, the literature has highlighted how the political environment influences these health determinants. In this sense, evidence indicates that the global spread of neoliberal economic austerity policies is drastically increasing mental illness rates, decreasing life expectancy and increasing inequities in access to health services ^(^ ^3^ ^-^ ^5^ ^)^ . 

 In countries on the capitalist periphery, such as Brazil, this policy has been shaped by the rapid and gradual destruction of the organizational/coordination capacities of health systems, the precariousness of working conditions and the quality of care. In the central countries of capitalism, neoliberal policy has incorporated the logic of eliminating restrictions on the market, with the rapid reduction, or even extinction, of public health care mechanisms ^(^ ^3^ ^-^ ^10^ ^)^ . 

 In addition, neoliberal policies have led to a dismantling of workers’ organizations and bargaining power, with an alarming growth in informality and high-cost, ineffective private health plans ^(^ ^4^ ^)^ . It is in this socio-political and epidemiological context that nurses construct their care for users in crisis situations, the most striking expression of mental health problems ^(^ ^1^ ^,^ ^4^ ^-^ ^11^ ^)^ . 

 Crisis situations in mental health can be understood in different ways. In the asylum model, it is defined as an acute situation or condition always resulting from a mental disorder, associated with the individual’s refusal of interventions and/or contact, personal impossibility of coping with the need for immediate intervention to reduce symptoms ^(^ ^12^ ^-^ ^13^ ^)^ . 

 On the other hand, the psychosocial model defines crisis as a social product with psychological repercussions, associated with the ability of individuals to respond to unbearable situations. It is related to the individual’s psychosocial repertoire, the result of a complex interaction between individual history, the individual’s behavior and the social/cultural environment ^(^ ^11^ ^,^ ^14^ ^-^ ^15^ ^)^ . 

 The psychosocial model of care is characterized as a specific way of combining techniques, technologies and organizational arrangements to address health problems, seeking to overcome the asylum model through community practices in an open environment and for active subjects. In this model, nurses have various tools at their disposal to develop their work and promote care in mental health crisis situations ^(^ ^16^ ^-^ ^17^ ^)^ . 

 Among the actions that this professional can carry out, the following stand out: using a welcoming approach, developing a bond through communication/listening skills, carrying out a physical health assessment, identifying social and behavioral risk conditions, creating joint care plans, taking part in psychoeducation actions, conducting and coordinating therapeutic groups, building permanent education actions, promoting the linking and monitoring of users to the appropriate points in the network. They can also advise on the correct use, benefits, side effects, duration and importance of adherence to pharmacological treatment ^(^ ^12^ ^-^ ^13^ ^,^ ^15^ ^,^ ^18^ ^)^ . 

 For these tools to be used, the literature highlights the need for quality mental health care to be provided by Primary Health Care (PHC), a strategy recognized worldwide for increasing access, reducing gaps in care and promoting comprehensive, longitudinal and effective care, and for it to be integrated into a network of services ^(^ ^19^ ^)^ . 

 To this end, the Psychosocial Care Network ( *Rede de Atenção Psicossocial* , RAPS) was created in Brazil, an organizational arrangement of mental health actions and services guided by the psychosocial care model. Despite the difficulties, the RAPS has led to an increase in investment and population coverage rates. However, even today around 77% of the Brazilian population still lives in regions with little or no RAPS assistance ^(^ ^20^ ^-^ ^22^ ^)^ . 

 In addition, the precarious material working conditions of nurses, the poor articulation and integration of the RAPS devices, including PHC, have led to the production of care with little resolution and lines of care incapable of covering mental health needs in their entirety ^(^ ^19^ ^,^ ^21^ ^-^ ^24^ ^)^ . 

In this way, it is important to know how the work of nurses assisting users in mental health crisis situations is structured, in other words, what objective/material and subjective conditions are present in nurses’ daily lives.

In view of the above, the question arises: Is the work carried out by nurses when dealing with individuals in a mental health crisis situation being developed to take into account all the possibilities they have in PHC? This research is based on the thesis that no, proposing that the professional’s work is restricted to a practical-technical approach focused on psychiatric diagnostic categories, and is therefore carried out in an alienated and fetishized way.

 To this end, this study’s results were interpreted using historical-dialectical materialism (HDM) and behavior analysis (BA). For HDM, nurses’ work consists of concrete work, that which is carried out and historically constructed to meet health needs, thus producing use values. For CA, the professional’s work is seen as operant behavior (apprehended) historically determined by a cultural community capable of instructing, replicating and modeling a myriad of classes of complex behaviors ^(^ ^14^ ^,^ ^25^ ^)^ . 

 There is a gap in the production of empirical evidence on mental health nursing work in Primary Health Care, especially in crisis situations, with a critical foundation and and using validated instruments such as the Theorical Domains Framework (TDF). This is especially true in Brazil, where the instrument’s implementation in this area is still small and in urgent need of identifying barriers, given the significant increase in mental health problems worldwide ^(^ ^1^ ^,^ ^26^ ^)^ . 

Thus, the study aimed to analyze the nurses’ work in PHC in the face of mental health crisis situations, based on the verbal reports of these professionals.

## Method

### Type of study and theoretical-methodological design

This is a descriptive-exploratory study with a qualitative approach, using CA and HDM as theoretical-interpretive frameworks and TDF as a methodological-analytical framework.

 The TDF is an instrument composed of a set of 14 domains and their respective constructs and aims to help identify the influences and barriers (“cognitive”, environmental and social) that determine a behavior. It suggests the following stages: 1) selection and specification of the target behavior — the nurse’s care work; 2) study design selection; 3) participant selection strategies; 4) interview script development; 5) data collection and analysis. The Consolidated Criteria for Reporting Qualitative Research (COREQ) was used to review and write the report ^(^ ^26^ ^-^ ^27^ ^)^ . 

 Key concepts from the interpretative theoretical frameworks were used for interpretation and synthesis. As for CA, the concepts of learning history, historical determinants of behavior (phylogeny, ontogeny and culture), positive and negative reinforcing events and reinforcement patterns were used. In relation to the HDM, the concepts of work (concrete and abstract), value (use and exchange), capital, merchandise, alienation, class conflict, production relations, exploitation, ideology, precariousness, neoliberalism, centre/periphery relationship and fetish were used ^(^ ^14^ ^,^ ^25^ ^)^ . 

### Setting

The study was carried out in six Basic Health Units (BHU) and six Family Health Units (FHU) in a capital city in Brazil’s Midwest region. The municipality’s healthcare network comprised 61 FHUs and 11 BHUs, distributed across seven health regions.

### Period

Data was collected between October 2022 and February 2023.

### Selection criteria

 The Who (nurses), When (user contact in a mental health crisis situation), Where (Primary Health Care) and How (practices and actions associated with nurses’ work) strategies were used to select and specify the behavior ^(^ ^26^ ^)^ . 

The study population was defined as: nurses with an employment contract and working in the FHUs and BHUs. In order to select the participants, the following inclusion criteria were stipulated: to be a nurse, to be employed and to have worked as a nurse in the FHU or BHU for at least six months.

The criterion of length of service was chosen arbitrarily by the authors, by means of a working group discussion, taking into account the time needed for the professional to get used to the work context and get to know their community. Professionals on leave and/or on vacation were excluded.

### Participants

 One nurse from each health unit took part in the study. Each FHU and BHU had a mean of two professional nurses. The researchers opted for one professional per unit so that the interviews would have a greater diversity of locations/units, without reaching the saturation criterion of 10 interviews followed by three additional ones, or until there were no new themes ^(^ ^28^ ^)^ . 

As a strategy for selecting participants, the units were divided into seven groups (G1 to G7) and the BHUs and FHUs in each health region were numbered (G1: BHU1; G2: BHU2; G1: FHU1; G2: FHU1 and so on).

Each unit group was then entered into an online draw tool to select one BHU and one FHU from each health region. The unit drawn was excluded if all the professionals in the unit refused. A total of fourteen health units were initially drawn, of which seven were BHUs and seven FHUs. Of these, only one was known to the main author of the research. In this case, the interview was conducted by another researcher.

A total of thirteen nurses agreed to take part in the study, but one was excluded due to difficulties in receiving the researchers on the day/place scheduled for the interview, after three attempts. A total of twelve professionals from the city’s seven health regions took part in the study.

### Data collection

Data was collected through a single semi-structured, individual, face-to-face interview, recorded on audio media, using a digital recorder, without image recording, lasting a mean of forty-five minutes.

A semi-structured script was used with questions about the professionals’ knowledge, strategies and skills used, intentions and goals, effectiveness of management, the professional’s role, social and professional support, barriers, facilitators and feelings. An instrument was also used to characterize the participants with age, marital status, time since graduation, color/ethnicity, workload, time in post and income.

Data collection was carried out by two researchers, an undergraduate student and a nurse (studying for a master’s degree), both trained beforehand.

### Data collection procedures

To begin data collection, the person in charge of the unit was contacted by telephone, and a time was agreed for the research to be presented, so as not to jeopardize the routine and care provided to the population.

During this visit, the researchers responsible for data collection presented the research proposal and data collection procedures to all the nurses at the unit.

After this initial contact with the nurses, three types of responses were obtained: 1) no agreement to take part; 2) agreement to schedule an interview; 3) agreement to carry out the interview at that time. In the first case, the unit was excluded from the list and a new draw was made within the group of the unit visited. Among the reasons cited by professionals for refusing to take part was the workload and the unavailability of colleagues to replace the professional during the interview.

In the second situation, the interview was scheduled for a day/place chosen by the participant, who was informed that he could cancel or reschedule at any time. When the participant’s situation prevented the interview from taking place, a new date was scheduled, within seven days of the initial date.

In units where more than one professional per unit expressed an interest in taking part, the researchers provided new guidance on the methodology and the professional was placed on the reserve list for use in situations where it was impossible to conduct the interview with the initially selected professional. In the third situation, the interview was carried out at the time of acceptance.

### Data treatment and analysis

All the audios of the interviews were transcribed in full, and the identifications of the participants, people and institutions mentioned were replaced by codes that only the researchers could use. Each interview was assigned a code describing the order of the interview and the group to which it belonged (e.g. G1:E1, G2:E2, G3:E3, and so on).

 The TDF instrument ( Figure 1 ) provided a structure of the determining factors (the domains and constructs) for nurses’ work in mental health crisis situations, i.e. facilitating the identification of the determinants that establish, maintain and modify behavior ^(^ ^26^ ^)^ . 

**Figure 1:** - Theorical Domains Framework: its domains, definition and constructs

**Domain**	**Definition**	**Construct**
1. Knowledge	Awareness of the existence of something.	-Knowledge (including knowledge of the condition/scientific basis) -Procedural knowledge -Knowledge of the task context
2. Capability	A skill or proficiency acquired through practice.	-Skills development -Competence -Ability -Interpersonal skills -Practice -Ability assessment
3. Identity and professional/social role	A set of coherent behaviors and the display of personal qualities of an individual in a social or work environment.	-Professional identity -Professional role -Social identity -Identity -Professional boundaries -Professional confidence -Group identity -Leadership -Organizational commitment
4. Beliefs about capabilities	Acceptance of the truth, reality or validity of a skill, talent or facility that a person can use constructively.	-Self-confidence -Perceived competence -Self-efficacy -Perceived behavioral control -Beliefs -Self-esteem -Empowerment -Professional confidence
5. Optimism	The confidence that things will turn out for the best or that the desired goals will be achieved.	-Optimism -Pessimism -Unrealistic optimism -Identity
6. Beliefs about consequences	Acceptance of the truth, reality or validity of the results of a behavior in a given situation.	-Beliefs -Result expectations -Result expectations characteristics -Anticipated regret -Consequences
7. Reinforcement	Increasing the probability of a response by establishing a relationship of dependence or contingency between the response and a given stimulus.	-Rewards (proximal/distal, with value/without value, probable/improbable) -Incentives -Punishment -Consequences -Reinforcement -Contingencies -Sanctions
8. Intentions	A conscious decision to perform a behavior or a determination to act in a certain way.	-Intent stability -Stages of change model -Trans-theoretical model and stage of change
9. Goals	Mental representations of results or final states that an individual wishes to achieve.	-Goals (distal/proximal) -Goal priority -Goals/goal setting -Goals (autonomous/controlled) -Action plan -Intention to implement
10. Memory, attention and decision-making processes	Ability to retain information, selectively focus on aspects of the environment and choose between two or more alternatives.	-Memory -Attention -Attention control -Decision making -Cognitive overload/tiredness
11. Environmental context and resources	Any circumstance in a person’s situation or environment that discourages or encourages the development of skills and abilities independence, social competence and adaptive behavior.	-Environmental stressors -Material resources -Organizational culture/climate -Salient events/critical incidents -Person x environment interaction -Barriers and facilitators
12. Social influences	Those interpersonal processes that can cause individuals to change their thoughts, feelings or behavior.	-Social pressure -Social norms -Group conformity -Social comparisons -Group norms -Social support -Power -Conflict between groups -Alienation -Group identity -Modeling
13. Emotion	A complex reaction pattern, involving experiential, behavioral and physiological elements, by which the individual attempts to deal with a personally significant issue or event.	-Fear -Anxiety -Affection -Stress -Depression -Positive/negative affections -Burnout
14. Behavioral regulation	Anything intended to manage or change objectively observed or measured actions.	-Self-monitoring -Breaking habits -Action plan

Source: Taken from the Theorical Domains Framework user guide ^(^ ^26^ ^)^

 The analysis process used the deductive method, involving five phases: 1) reading and exploring the material; 2) coding and pairing the content of the speeches into the constructs and domains that best reflected the transcribed content; 3) absolute and proportional distribution of the number of speeches; 4) elaboration of the themes considering the intersections of the speeches and the interpretative theoretical references; 4) construction of a map illustrating the relationships between the themes/domains ^(^ ^26^ ^,^ ^29^ ^)^ . 

 This process was carried out independently by two researchers trained in the use of the instrument and the analysis was then compared and discussed until consensus was reached. When consensus could not be reached, the speech/interview excerpt was allocated to all the domains listed by the researchers ^(^ ^26^ ^)^ . 

All this analysis was guided by the construction of tables containing the participants’ statements. The statements were interpreted based on the construct definitions described in the TDF and against the theoretical frameworks of CA and HDM. The data characterizing the sample and the quantitative distribution (absolute and proportional) were organized and analyzed in an Excel document (Microsoft).

### Ethical aspects

The study was approved by the Ethics Committee for Research Involving Human Beings, according to CAAE: 59722622.9.0000.0021.

## Results

 A total of twelve professionals took part in the study, seven of whom were male (58.3%), with a mean age of 33 years (maximum 47, median 31.5 and minimum 24), most of whom were single (58.3%), eight (66.7%) identified themselves as brown, two (16.7%) as white and one as black (8.3%). Time in post varied between one and ten years, with a mean of three and a half years. The mean family income of the professionals was R$8,550 *reais* . 

 The analysis identified constructs from the 14 domains, with the following distribution ( Table 1 ). 

**Figure 2:**
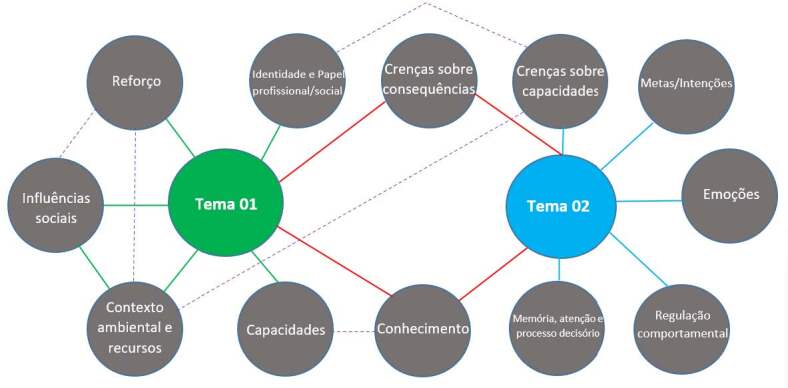
- Map of themes and intersections between TDF* domains

 After analysis and interpretation, the data was grouped into two themes: “Nurses’ work: material and social determinants (THEME-01)” and “Nurses’ subjective conditions in mental health crisis situations (THEME-02)” (see Figure 2 ). 

**Table 1:** - Absolute and proportional distribution (by domain and in relation to the total) of the constructs and domains. Campo Grande, MS, Brazil, 2022-2023

**Number of events**	Proportion by domain (% ^*^ )	**Construct**	**Domain**
61	14.48	Knowledge (including knowledge of the condition/scientific basis)	Knowledge
74		Procedural knowledge	
97		Knowledge of the task context	
44	17.01	Resources/material resources	Environmental context and resources
3		Environmental stressors	
15		Organizational culture/climate	
96		Barriers and facilitators	
10		Salient events/critical incidents	
98		Person vs. environment interaction	
9	14.61	Capability	Capabilities
15		Capacity development	
59		Competence	
20		Interpersonal skills	
31		Practice	
55		Capacity assessment	
42		Skills	
49	9.55	Professional identity	Identity and professional/social role
65		Professional role	
3		Leadership	
34		Group identity	
10	8.16	Self-confidence	Beliefs about capabilities
5		Perceived competence	
6		Self-efficacy	
43		Beliefs	
3		Empowerment	
44		Perceived Behavioral Control	
18		Professional confidence	
23	7.21	Beliefs	Beliefs about consequences
25		Results Expectations	
15		Characteristics of result expectations	
9		Early regret	
42		Consequences	
1	1.20	Identity	Optimism
7		Optimism	
9		Pessimism	
2		Unrealistic optimism	
17	14.17	Reward (proximal/distal, valid or not valid, probable or improbable)	Reinforcement
1		Incentives	
49		Punishment	
43		Consequents	
21		Reinforcement	
92		Contingencies	
1		Sanctions	
8	0.51	Stability of intent	Intentions
17	3.92	Goals (distal/proximal)	Goals
5		Goals (autonomous/controlled)	
13		Goal/Goal setting	
13		Prioritizing goals	
12		Implementation intentions	
2		Action plan	
4	2.97	Social pressure	Social influences
1		Social comparisons	
1		Group conformity	
1		Social norms	
37		Social support	
2		Conflict between groups	
1		Power	
2	3.35	Fear	Emotions
1		Anxiety	
27		Affection	
0		Stress	
17		Positive/negative affections	
6		Exhaustion	
13	0.82	Self-monitoring	Behavioral regulation
1		Breaking habits	
18		Action plan	
1	2.02	Memory	Memory, attention and decision-making
11		Attention control	
8		Attention	
11		Decision-making	
1		Cognitive overload/tiredness	

^*^ Percentage value of the proportional (relative) distribution of each domain

*Theorical Domains Framework

Note: Green line: single relationship with Theme 01; blue line: single relationship with Theme 02; red line: relationship between the two themes; dashed line: relationship between constructs; qwn elaboration, based on literature ^(^ ^29^ ^)^

### Nurses’ work: material and social determinants

 The activities related to the nurse’s care work were reported in the domains of capacity and procedural knowledge, with practices such as: referring users to the Urgent and Emergency Service ( *Serviço de Urgência e Emergência* , SUE) or specialized services such as Centers of Psychosocial Attention ( *Centros de Aten* ção *Psicossocial* , CAPS), Family Health Support Centers ( *Núcleos de Apoio à Saúde da Família* , NASF); hospitalization; risk classification; medication and/or mechanical restraint; reception, home visits in exceptional cases and active search. 

*(...) there are two alternatives, one is to call urgent and emergency care so that they can restrain them, so that they don’t self-harm [...].* (E1) 

*(...) when he comes into the unit, he is heard, if it’s obvious that he’s in a moment of crisis [...] he’s treated as an emergency, the highest classification here. [...] he’ll be identified and sent to the nurse’s office for a risk classification [...] we refer patients via the vacancy system [...].* (E08) 

*(...) we refer them to the CAPS, because they’ll be assessed there, and I’ll transfer them to a psychologist, sometimes a psychiatrist, and if necessary, hospitalization.* (E10) 

*Welcoming. People need attention and affection, and to understand that someone is looking out for them, without judging. So, I think this is the key point of Primary Care [...] welcoming the user within their individuality.* (E09) 

The nurse is described (identity and professional/social role) as the professionals with the most contact with users, responsible for welcoming, supporting and dialoguing, and for classifying/organizing care. They were also cited as being responsible for liaison between the team, the family and with RAPS components.

*Nursing is the first contact when I’m talking to the patient [...] but we have more, let’s say, familiarity even with the families [...] And giving emotional support too, we have this role of treating the patient’s emotions, offering support.* (E02) 

*If the patient is not stable, we try to stabilize him here, he goes through the nursing consultation, classification and then he is sent to the doctor who stabilizes the condition with some medication [...].* (E08) 

*Look, I see the nurse as someone who is going to organize the care, direct it well [...].* (E10) 

*We survey demand and it’s the nurse who articulates this process, including with the family, since we’re the ones who are going to be at the house to provide guidance and to be able to bring in the multidisciplinary team to discuss the cases.* (E03) 

*It is also our role, as I can tell you, in trying to resolve these cases. [...] ... for this condition that the patient has been presenting.* (E05) 

The actions and practices carried out in PHC, according to the professionals, have consequences for adherence to treatment (improvement/ worsening); difficulties in following up and identifying cases; breaking the bond and recurrence of crises.

*And the other thing is adherence. Adherence to treatment. So, the patient thinks they’re fine and then they don’t adhere to the treatment anymore and then the crisis comes again. [...].* (E03) 

*So, when we use this method of care, which is welcoming, listening, we end up with very high patient adherence [...]. When they are able to perceive the health and illness process, this makes it easier for them to adhere to treatment [...] it helps them to change their lifestyle [...].* (E05) 

*(...) so the patient doesn’t bond with their team doctor, they don’t bond with the nurse who represents the whole team. [...] so how can the patient create a bond if every time they come here it’s a different doctor? [...].* (E08) 

In relation to the environmental and resource context, the nurses reported: an insufficient number of vacancies, difficulties in accessing services (long waiting times, for example), scrapping of structures and adequate spaces for care, insufficient human resources and high turnover. This context has been linked to consequences such as professional overload and a reduction in the number of professionals available to care for users.

*(...) there’s a lot of wear and tear on the forty-hour professional, there’s a lot of suffering within this process with all this load [...].* (E09) 

*(...) “And my concern also sometimes relates to the shortage of places available for the timely treatment of these patients.”* (E02) 

*(...) then the person gets in a queue, takes 5 to 6 months to get an appointment [...].* (E01) 

*(...) the Unified Health System (SUS) overload, the system overload itself and the lack of professionals, the scrapping of the SUS that is happening in the country [...].* (E04) 

*Sometimes there is not enough room in the physical structure, it’s one room for four nurses, so there’s often nowhere to take the patient to talk to them better.* (E12) 

In relation to social influences (social support, social pressure, conflict between groups), the presence of support for referral to specialized services (CAPS and NASF) and Urgent and Emergency Services (SUE) was described, but with compromised communication and counter-referral. Interference from the medical team in the nurse’s autonomy was also mentioned.

*(...) there’s no conversation, there’s no counter-referral, there’s not that certain autonomy that I’m going to send the patient and have access to everything that’s being done [...]. So, it’s an endless loop where we have to keep going back, there’s no such thing [...].* (E08) 

*(...) And I can call the fire brigade, I can call the Mobile Emergency Care Service (* Serviço de Atendimento Móvel de Urgência, SAMU *) too [...].* (E02) 

*(...) because I don’t have access, or my category doesn’t allow it, or what I can do is sometimes dismantled by another professional, by the medical issue [...].* (E12) 

Monthly education/discussion activities were also reported as a form of social support.

*Every month we have monthly meetings at the Municipal Specialized Center (* Centro Especializado Municipal, CEM *) with the mental health team, where we discuss various topics relating to patient flow and regulation demands. We discuss cases, we take real cases that happen in the units to discuss to see what we could have done, we list the strengths and weaknesses of this service.* (E05) 

### Nurses’ subjective conditions in mental health crisis situations

Nurses reported insecurity/lack of technical capacity, as well as situations where stigma was expressed towards users.

*(...) the lack of technical capacity [...] I try my best as a nurse, based on everything I’ve learned, but am I an expert in this? No! Am I part of a group that always has continuing education on this so that I know how to handle cases? No!* (E08) 

*(...) Yes, there are some prejudices of a religious nature, social nature, economic nature [...] I think that one of the factors is still discrimination against mental patients. They often say it’s demonic possession [...] they say the patient is a “vagabond”.* (E12) 

Mental health crisis (knowledge of the condition) was associated with the notion of psychiatric urgency/emergency, and the term “outbreak” was sometimes used. In addition, when specifying the types of crisis, participants mentioned signs/symptoms of mental disorders.

*The crisis is the psychiatric outbreak, I consider it to be a situation in which the individual is out of their mind, speaking in terms of urgency [.…].* (E02) 

*It can be anything from an anxiety crisis, a panic crisis, patients in an outburst due to some kind of disorder, bipolar disorder, schizophrenic patients in some kind of outburst [...].* (E06) 

As an exception, there were two reports that described an understanding of the crisis that was more in line with the psychosocial model.

*So, I understand the mental health crisis as a process [...], but something unexpected that the patient is unable to cope with on their own, or some external event [...].* (E04) 

*(...) I think it’s all those problems in which the person is no longer able to have good mental and psychological judgment, which ends up interfering in daily activities, both in terms of family, friends and work. It ends up compromising the person’s social ties... [...]* (E11) 

In relation to beliefs (capacities and consequences), the participants reported believing that they reduce the risks, but that their work does not help to treat the disorders, but only controls the crises occasionally.

*(...) not that it avoids a major problem, but we manage to minimize the risks of the severity of the disease or of an outbreak itself or of attempted suicide [...] a reduction in harm [...].* (E02) 

*(...) the cause of the problem hasn’t been solved, that triggering event sometimes hasn’t even been solved [...] I think our care today is making patients addicted to medication, it’s not helping to control the disease in any way.* (E06) 

They point out that their work (goals) is aimed at: reducing suffering, adapting and regularizing medication treatment, linking users to specialized services and stabilizing them so that they can return to society.

*Then, the biggest goal is to reduce the patient’s psychological suffering, because it’s no use having a zero queue, but I have users suffering absurdly from psychological pain, it hurts more than physical pain* (E09). 

*In fact, when we use these strategies, we try to bring patients back to regular treatment [...].* (E07) 

*When it comes to depressed people, preventing them from reaching the stage of self-harm, self-mutilation, self-extermination [...]. Make a referral to CAPS, which is the Psychosocial Support Center we have in the city.* (E11) 

*Trying to stabilize them so that they can live in normal society [...] ...with the intention of getting them back into social life [...]* (E03). 

Finally, the nurses reported emotions of anguish, fear, unpreparedness/inability, frustration, guilt and tiredness.

*Frustration, I think, is the strongest. [...]. Then comes the frustration of not being able to do more than that to help.* (E11) 

*So, this generates a lot of anguish in the professional [...].* (E09) 

*A lot of professionals don’t have the capacity, they’re afraid, they don’t know how to act, often at the moment of a crisis [...].* (E12) 

*But I don’t feel that way, I don’t enjoy meeting this kind of demand, you know? It’s not something that catches my attention.* (E05) 

## Discussion

The results reinforce the thesis that nurses’ work is restricted to the “technical” sphere focused on psychiatric diagnoses, permeated by objective and subjective contradictions that interfere directly or indirectly in the strategies used in their work process and in caring for individuals in situations of mental health crisis.

 Thus, even with the tools and technologies for nurses to carry out a job that promotes a positive experience of the crisis, the conditions and resources available end up stimulating a protocol and exhaustive execution, given the professional overload, of fixed care stages; risk classification, identification of signs/symptoms and directing users to specialized services; which, directly or indirectly, promote the phenomenon of medicalization and pathologization of mental health problems ^(^ ^12^ ^,^ ^16^ ^,^ ^30^ ^)^ . 

 In addition, working conditions increase the difficulties in organizing continuing education strategies, which are essential for developing adequate skills to deal with health conditions; a situation identified both in this study and also seen in national and international literature ^(^ ^16^ ^,^ ^30^ ^)^ . 

 In this sense, it can be assumed that a manicomial perspective persists in PHC, supported by a neoliberal project of political-economic asphyxiation, based on austerity, which in a country like Brazil is configured by the gradual reduction of the state and its capacity to implement public health policies ^(^ ^5^ ^,^ ^21^ ^-^ ^24^ ^)^ . 

 For the HDM, this project is essential for transforming mental health and the crisis into a commodity. To do this, the experience of human suffering needs to be “simplified” and placed within pre-established diagnostic criteria, a procedure that makes it easier to direct demands to the pharmaceutical industry, private and/or religious health networks, and therapeutic communities ^(^ ^25^ ^,^ ^31^ ^-^ ^34^ ^)^ . 

 In addition, PHC nurses, along with users and their colleagues, have to live with an environment of precariousness, marked by overload, low resolution, limited services supply, high turnover, difficulty in building bonds, instability of employment relationships and incentives for flexibilization/outsourcing ^(^ ^21^ ^-^ ^23^ ^,^ ^30^ ^-^ ^32^ ^)^ . 

 In this way, nurses’ work, which is essentially concrete and endowed with intrinsic use value, ends up becoming alienated from its social function, the production of health through care, and is constituted only as a workforce that carries out standardized activities, integrating the chain of capital appreciation (value that generates value) indirectly, speeding up the circulation and exchange of goods ^(^ ^25^ ^,^ ^33^ ^)^ . 

 Furthermore, as seen in the study, sometimes the understanding of mental health becomes indistinguishable from psychiatric diagnoses, acting only as its negative (“not having a disorder”), and the crisis is seen as the severe acute situation of a mental disorder; a phenomenon also present in the literature ^(^ ^30^ ^-^ ^33^ ^)^ . 

 This is the socialization of a technical fetish, in other words, the subjective acquires an objective character, being simplified, easily identified, categorized and controlled; suffering acquires a form that is more conducive to market dynamics. Psychiatry thus acquires social and coercive power, decontextualizing behavior from the individual’s environment (current and previous), from reinforcing and aversive variables and from each individual’s personal learning history ^(^ ^14^ ^,^ ^25^ ^,^ ^31^ ^-^ ^32^ ^)^ . 

 In other words, visible (public) behaviors and (private) emotions are now explained by mental disorders, and all life history — violence, neglect, abuse, etc. — is treated, at most, as “non-modifiable individual risk factors”. This form of “explanation” is validated and shared on a daily basis by the ideological capital apparatus, given the constant need to manage the products (suffering) of the contradictions of exploitative labor relations. Capital therefore profits from the suffering it generates, and furthermore, individualizes the determinants of this suffering, blaming individuals for the social ills of the capitalist mode of production ^(^ ^14^ ^,^ ^25^ ^,^ ^31^ ^-^ ^32^ ^)^ . 

 The understanding of mental health and the precarious conditions faced by professionals may also explain why actions that would theoretically be associated with psychosocial care, such as welcoming and bonding, become stages in a “technical” process, stripped of their humanizing character, a criterion that requires “spending different amounts of time compared to conventional demands” ^(^ ^11^ ^,^ ^15^ ^,^ ^18^ ^,^ ^31^ ^)^ . 

 This panorama, it should be emphasized, is not exclusive to peripheral countries such as Brazil, but is present in all countries, to a lesser or greater extent. For example, in the United States of America, the lack of a public health system and growing social inequalities have disproportionately affected the most vulnerable population, with an alarming increase in homelessness, mass incarceration and worsening health conditions ^(^ ^5^ ^)^ . 

 In China, despite efforts to reduce the inequity of access to health care, the decrease in the number of mental health workers, including nurses, and the difficulties in training these professionals have brought challenges to the organization of a community mental health system. A similar situation can be found in other countries in Europe and the Middle East ^(^ ^6^ ^,^ ^10^ ^,^ ^35^ ^-^ ^37^ ^)^ . 

 In addition, it is noteworthy that, both in Brazil and in other countries, nursing professionals themselves have to deal with the inequities of social markers of gender and race; the majority of the nursing workforce is made up of women, and in Brazil, of black women who face double/triple working hours ^(^ ^5^ ^-^ ^6^ ^)^ . 

 The emotional and affective repercussions (frustration, fear, etc.) reported by nurses are not surprising. Given the limited possibilities and actions, low social support, lack of continuing education, poor resolution and increasingly precarious living conditions, the professional’s feeling of frustration is justifiable, given that emotions are reactions to the environment (learned reflex behavior) ^(^ ^14^ ^)^ . 

 In this sense, whether or not a professional persists in building new strategies, trying new possibilities, looking for new communication networks, among others, is related to a specific reinforcement pattern in the professional’s history, in other words, the reason and intervals that led the professional to try alternatives and whether or not they were successful. For example, the number of times a professional has tried to build a singular therapeutic project with the NASF, based on the number of successes obtained ^(^ ^14^ ^)^ . 

 Furthermore, the emotional/psychological repercussions can also be found in other countries. Nurses have faced situations of violence, moral harassment, bullying from co-workers and organizational problems that undermine the effectiveness of their care. International literature has revealed a complex scenario of illness at work which sometimes leads to outcomes such as suicide ^(^ ^37^ ^)^ . 

This makes it problematic to hold nurses alone responsible for the results of mental health crisis care, since there are no concrete conditions for building actions related to the psychosocial model.

 In this sense, individualizing the problem would only increase the number of situations capable of reinforcing “negative” emotions; frustration/blame. Added to this is the weakness in the systematic structuring of continuing education actions, the overload, the low availability of time and inadequate resources that make it unlikely to systematically apply the best care strategies to mental health crisis situations ^(^ ^14^ ^,^ ^16^ ^,^ ^30^ ^-^ ^31^ ^)^ . 

 There is no doubt, and the literature reinforces this point exhaustively, about the need to invest in training and continuing education for nurses to deal with crisis situations. However, for investments to be possible, and for there to be concrete conditions favorable to the development of psychosocial care by nurses, the state and its population need to overcome neoliberal politics, completely banishing the merchandise logic from the health field ^(^ ^5^ ^,^ ^16^ ^,^ ^31^ ^-^ ^32^ ^,^ ^37^ ^)^ . 

As a category with strong social power, which lives in and has daily contact with its community, nurses can contribute to this struggle by strengthening their political and social role and becoming more involved in the struggle of the working class. They should therefore be active through unions, councils, vanguard parties, social movements and initiatives, fighting for fair working conditions and overcoming poverty, lack of housing, sanitation, decent work, among other problems that plague capitalist society.

In relation to the study’s limitations, it should be noted that PHC is just one of the RAPS settings where nurses can provide care to individuals in mental health crisis situations. It is therefore vital that further studies are carried out so that the whole context of the RAPS can be analyzed in its entirety, in a process that involves various categories of health professionals and other actors, including users.

## Conclusion

In summary, the study made it possible to analyze how nurses’ work in PHC is structured in the face of mental health crisis situations, describing its objective and subjective contradictions — a mostly asylum-based understanding and a work surrounded by the phenomenon of alienation and fetishization of “technique”, which serves to indirectly insert concrete health work into the chain of capital appreciation.

Nurses are aware that their care in crisis situations can be carried out in another way, but given the overexploitation and precariousness of resources, there are few conditions left for this construction, implying emotional/affective repercussions of frustration, fear, among others, on the professionals.

Finally, the application of critical references, as well as the methodological systematization made possible by the TDF instrument, is vital in this situation, as it clarifies the need for a greater theoretical and scientific understanding of the social and material conditioning factors of health work.
