# Supplementary material for: Mental health crisis situations: the nurse’s work in Primary Health Care
Source: Rev Lat Am Enfermagem. 2024 Oct 25;32:e4356. doi: 10.1590/1518-8345.7015.4356 (PMC11526249; doi:10.1590/1518-8345.7015.4356)
Supplement: Translation

# Translation: Situaciones de crisis de salud mental: el trabajo del enfermero en la Atención Primaria de Salud ^*^

**DOI:** 10.1590/1518-8345.7015.4355

**Authors:** Gessner Bravo de Paula, Nur Mohamad Ali El Akra, Lucas Ferraz Córdova, Lucilene Cardoso, Ana Carolina Guidorizzi Zanetti, Bianca Cristina Ciccone Giacon Arruda

**Affiliations:** ^1^ Universidade Federal do Mato Grosso do Sul, Instituto Integrado de Saúde, Campo Grande, MS, Brasil.; ^2^ Secretaria do Estado de Saúde, Coordenadoria Estadual do Telessaúde, Campo Grande, MS, Brasil.; ^3^ Becario de la Fundação de Apoio ao Desenvolvimento do Ensino, Ciência e Tecnologia do Estado do Mato Grosso do Sul (FUNDECT), Brasil.; ^4^ Becario de la Coordenação de Aperfeiçoamento de Pessoal de Nível Superior (CAPES), Brasil.; ^5^ Universidade Federal do Mato Grosso do Sul, Faculdade de Ciências Humanas, Campo Grande, MS, Brasil.; ^6^ Universidade de São Paulo, Escola de Enfermagem de Ribeirão Preto, Centro Colaborador de la OPS/OMS para el Desarrollo de la Investigación en Enfermería, RibeirãoPreto, SP, Brasil.

**Keywords:** Atención Primaria de Salud, Enfermeras y Enfermeros, Intervención en la Crisis (Psiquiatría), Modelos de Atención de Salud, Salud Mental, Trabajo

## Abstract

**Objetivo:**

analizar el trabajo del enfermero en la Atención Primaria de Salud en situaciones de crisis de salud mental.

**Método:**

estudio descriptivo-exploratorio, con enfoque cualitativo, basado en los marcos teórico-interpretativos del análisis de la conducta y el materialismo histórico-dialéctico. Los datos fueron recolectados a través de una entrevista semiestructurada a doce enfermeros de Atención Primaria de Salud y analizados mediante la técnica deductiva propuesta por el *Theorical Domains Framework* , marco metodológico adoptado.

**Resultados:**

los datos analizados permitieron elaborar dos temas: “El trabajo del enfermero: determinantes materiales y sociales” y “Condiciones subjetivas del enfermero en situaciones de crisis de salud mental”. El trabajo del enfermero estuvo guiado por la ejecución protocolar de actuaciones “técnicas” relacionadas con la clínica psiquiátrica, que considera la crisis como un “brote psiquiátrico”.

**Conclusión:**

el estudio permitió analizar el trabajo del enfermero en situaciones de crisis, al describir las contradicciones objetivas y subjetivas, la comprensión que tienen del fenómeno de la crisis y las repercusiones emocionales de ese trabajo en los profesionales. Se observó que el trabajo está permeado por la alienación y el *fetiche* de la técnica, lo que indica que es necesario que la categoría enfermería actúe, se organice y movilice político-socialmente en torno a la lucha contra el modelo de atención manicomial y a la consolidación de la perspectiva de la Reforma psiquiátrica.

## Puntos destacados

**(1)** Protocolo de atención manicomial para identificar y reducir los síntomas de la crisis.

**(2)** Se reduce la experiencia de la crisis a un fenómeno psicopatológico.

**(3)** Uso de marcos teórico-críticos que sigan las propuestas de la atención psicosocial.

**(4)** Marco metodológico-analítico validado internacionalmente.

**(5)** Es necesario que los enfermeros realicen trabajo político-social.

## Introducción

 El deterioro de la salud mental presenta un crecimiento significativo en todo el mundo en la población general. En 2019, se estimó que aproximadamente el 13% de la población mundial (970 millones de personas) tenía algún tipo de trastorno de salud mental, y que los más prevalentes eran los trastornos de ansiedad (31%), la depresión (28,9%) y los derivados del uso del alcohol y otras drogas ^(^ ^1^ ^)^ . 

 La Organización Mundial de la Salud define la salud mental como un estado en el que un individuo puede funcionar, prosperar, afrontar dificultades, conectarse y contribuir para mejorar su comunidad. Por lo tanto, son problemas estrechamente relacionados/determinados por condiciones físicas, biológicas, socioculturales y conductuales ^(^ ^2^ ^-^ ^4^ ^)^ . 

 Además, la literatura ha destacado cómo el entorno político influye en estos determinantes de la salud. La evidencia indica que la difusión global de la política de austeridad económica neoliberal está aumentando drásticamente las tasas de enfermedades mentales, reduciendo la esperanza de vida y aumentando las inequidades en el acceso a los servicios de salud ^(^ ^3^ ^-^ ^5^ ^)^ . 

 En países de la periferia del capitalismo, como Brasil, esta política se ha caracterizado por la rápida y gradual destrucción de las capacidades de organización/coordinación de los sistemas de salud, precarización de las condiciones laborales y de la calidad de la atención. En los países capitalistas centrales, la política neoliberal incorporó la lógica de eliminar las restricciones del mercado, con la rápida reducción, o incluso la extinción, de los mecanismos de asistencia pública a la salud ^(^ ^3^ ^-^ ^10^ ^)^ . 

 Además, la política neoliberal provocó una desarticulación organizacional y del poder de “negociación” de los trabajadores, que conlleva un crecimiento alarmante de la informalidad y de los planes de salud privados con altos costos y poca efectividad ^(^ ^4^ ^)^ . Es en este contexto sociopolítico y epidemiológico que los enfermeros construyen su atención a usuarios en situación de crisis, la expresión más llamativa de los problemas de salud mental ^(^ ^1^ ^,^ ^4^ ^-^ ^11^ ^)^ . 

 La situación de crisis de salud mental se puede entender de diferentes maneras. En el modelo manicomial, se define como una situación o condición aguda siempre derivada de un trastorno mental, asociada con el rechazo del individuo a intervenciones y/o contacto, incapacidad personal de afrontamiento que requiere intervención inmediata para reducir los síntomas ^(^ ^12^ ^-^ ^13^ ^)^ . 

 Por otro lado, el modelo psicosocial define la crisis como un producto social con repercusiones psicológicas, asociadas a la capacidad de los sujetos para responder a situaciones insoportables. Está relacionado con el repertorio psicosocial del individuo, producto de una interacción compleja entre la historia individual, el comportamiento individual y el entorno social/cultural ^(^ ^11^ ^,^ ^14^ ^-^ ^15^ ^)^ . 

 El modelo psicosocial de atención se caracteriza por ser una forma específica de combinar técnicas, tecnologías y convenios organizativos para abordar los problemas de salud, que busca superar el modelo manicomial a través de prácticas comunitarias en un ambiente abierto y para sujetos activos. En este modelo, el enfermero tiene a su disposición varias herramientas para desarrollar su trabajo y promover el cuidado en situaciones de crisis de salud mental ^(^ ^16^ ^-^ ^17^ ^)^ . 

 Entre las acciones que dicho profesional puede realizar se destacan: utilizar la recepción; desarrollar vínculos a través de habilidades de comunicación/escucha; realizar evaluaciones de salud física; identificar condiciones sociales y conductas de riesgo, crear planes de atención conjuntos; participar en acciones de psicoeducación, liderar y coordinar grupos terapéuticos; crear acciones de educación continua; promover el vínculo y seguimiento de los usuarios hasta los puntos adecuados de la red. También puede brindar orientación sobre el uso correcto, los beneficios, los efectos secundarios, la duración e importancia de la adherencia al tratamiento farmacológico ^(^ ^12^ ^-^ ^13^ ^,^ ^15^ ^,^ ^18^ ^)^ . 

 Para que esas herramientas se puedan utilizar, la literatura destaca que es necesario que la Atención Primaria de Salud (APS) brinde atención de salud mental de calidad; estrategia reconocida mundialmente para aumentar el acceso, reducir las brechas en la atención y promover una atención integral, longitudinal y efectiva, que esté integrada en una red de servicios ^(^ ^19^ ^)^ . 

 Para ello, se creó en Brasil la Red de Atención Psicosocial (RAPS), un convenio organizacional de acciones y servicios de salud mental orientados por el modelo de atención psicosocial. A pesar de las dificultades, la RAPS promovió el crecimiento de las tasas de inversión y cobertura poblacional. Sin embargo, aún hoy alrededor del 77% de la población brasileña todavía vive en regiones donde la asistencia de la RAPS es baja o nula ^(^ ^20^ ^-^ ^22^ ^)^ . 

 Además, las precarias condiciones materiales de trabajo de los enfermeros, la baja articulación e integración de los dispositivos de la RAPS, que incluye la APS, se han vinculado a una producción de cuidados con poca resolución y líneas de atención incapaces de contemplar las necesidades de salud mental de forma integral ^(^ ^19^ ^,^ ^21^ ^-^ ^24^ ^)^ . 

Por lo tanto, es importante conocer cómo se estructura el trabajo de atención del enfermero a los usuarios en situaciones de crisis de salud mental, es decir, cuáles son las condiciones objetivas/materiales y subjetivas presentes en el trabajo diario del enfermero.

Por consiguiente, surge la pregunta: ¿el trabajo que realiza el enfermero con personas en situación de crisis de salud mental se desarrolla contemplando todas las posibilidades de acción que tiene en la APS? Esta investigación parte de la tesis de que no lo hace, y propone que el trabajo del profesional se restringe a una acción práctica-técnica enfocada en categorías diagnósticas psiquiátricas, por lo tanto, se realiza de manera alienada y fetichizada.

 Por ello, los resultados de este estudio fueron interpretados mediante el materialismo histórico-dialéctico (MHD) y el análisis de la conducta (AC). Para el MHD, el trabajo del enfermero consiste en un trabajo concreto, realizado y construido históricamente para satisfacer las necesidades de salud, que produce valores de uso. Mientras que, en el AC el trabajo del profesional se considera una conducta operante (aprendida) históricamente determinada por una comunidad cultural capaz de instruir, replicar y modelar una infinidad de clases de conductas complejas ^(^ ^14^ ^,^ ^25^ ^)^

 Cabe destacar que hay una laguna en la producción de evidencia empírica sobre el trabajo de enfermería en salud mental en la Atención Primaria de Salud, especialmente en situaciones de crisis, con fundamentación crítica y uso de instrumentos validados, como el *Theorical Domains Framework* (TDF). Eso ocurre principalmente en Brasil, donde el instrumento sobre el tema se implementa poco y es necesario identificar de forma urgente cuáles son las barreras, considerando el significativo aumento de los problemas de salud mental a nivel mundial ^(^ ^1^ ^,^ ^26^ ^)^ . 

Por lo tanto, el estudio tuvo como objetivo analizar el trabajo del enfermero en la APS en situaciones de crisis de salud mental a partir de los relatos verbales de esos profesionales.

## Método

### Tipo de estudio y diseño teórico-metodológico

Se trata de un estudio descriptivo-exploratorio, con enfoque cualitativo, en el que se utilizó el AC y el MHD, como marcos teórico-interpretativos, y el TDF, como marco metodológico-analítico.

 El TDF es un instrumento compuesto por un conjunto de 14 dominios y sus respectivos constructos, cuyo objetivo es ayudar a identificar las influencias y barreras (“cognitivas”, ambientales y sociales) que determinan el comportamiento. Sugiere los siguientes pasos: 1) selección y especificación de la conducta objetivo, el trabajo de atención del enfermero; 2) selección del diseño del estudio; 3) estrategias de selección de participantes; 4) desarrollo de un formulario de entrevista; 5) recolección y análisis de datos. Para revisar y redactar el informe se utilizaron los *Consolidated Criteria for Reporting Qualitative Research* (COREQ) ^(^ ^26^ ^-^ ^27^ ^)^ . 

 Para la interpretación y síntesis se utilizaron conceptos clave de marcos teóricos interpretativos. En cuanto al AC, se utilizaron los conceptos de historia de aprendizaje, determinantes históricos de la conducta (filogenia, ontogenia y cultura), eventos reforzadores positivos y negativos y patrones de refuerzo. En lo que respecta al MHD, se utilizaron los conceptos de trabajo (concreto y abstracto), valor (uso e intercambio), capital, mercancías, alienación, conflicto de clases, relaciones de producción, explotación, ideología, precariedad, neoliberalismo, relación centro/periferia y fetiche ^(^ ^14^ ^,^ ^25^ ^)^ . 

### Escenario

El estudio se realizó en seis Unidades Básicas de Salud (UBS) y seis Unidades de Salud de la Familia (USF), de una capital de la Región Centro-Oeste de Brasil. La red de atención de salud de la ciudad contaba con 61 USF y 11 UBS, distribuidas en siete regiones sanitarias.

### Periodo

La recopilación de datos se realizó entre octubre de 2022 y febrero de 2023.

### Criterios de selección

 Para la selección y especificación de la conducta se utilizó la estrategia: *Who* (enfermeros), *When* (contacto del usuario en situación de crisis de salud mental), *Where* (Atención Primaria de Salud) y *How* (prácticas y acciones asociadas al trabajo del enfermero) ^(^ ^26^ ^)^ . 

La población de estudio fue definida como: enfermeros con vínculo laboral y que trabajan en la USF y la UBS. Para la selección de los participantes se estipularon los siguientes criterios de inclusión: ser enfermero, estar asignado y haber ocupado el cargo de enfermero en la USF o en la UBS durante al menos seis meses.

El criterio tiempo de trabajo fue elegido arbitrariamente por los autores, mediante una discusión en el grupo de trabajo teniendo en cuenta el tiempo necesario para que el profesional se acostumbre al contexto de trabajo y conozca su comunidad. Se excluyeron los profesionales que estuvieran de licencia y/o de vacaciones.

### Participantes

 Participó del estudio un enfermero de cada unidad de salud. Cada USF y UBS contaba, en promedio, con dos profesionales enfermeros. Los investigadores optaron por incluir a un profesional de cada unidad para que las entrevistas tuvieran una mayor diversidad de ubicaciones/unidades, sin llegar al criterio de saturación de 10 entrevistas seguidas de tres entrevistas adicionales o hasta que no hubiera presencia de nuevos temas ^(^ ^28^ ^)^ . 

Como estrategia de selección de participantes, las unidades se dividieron en siete grupos (G1 a G7) y se numeraron las UBS y USF de cada región sanitaria (G1:UBS1; G2:UBS2; G1:USF1; G2:USF1, etc.).

 A continuación, se ingresó cada grupo de unidades a una herramienta de lotería *online* , para seleccionar una UBS y una USF de cada región sanitaria. La unidad seleccionada era excluida si todos los profesionales de la unidad se negaban a participar. Inicialmente se sortearon catorce unidades de salud, siete UBS y siete USF. El autor principal de la investigación sólo conocía una de ellas. En ese caso la entrevista fue realizada por otro investigador. 

Fueron trece los enfermeros que aceptaron participar del estudio, sin embargo, uno de ellos fue excluido porque tenía dificultades para recibir, después de tres intentos, a los investigadores en el día/lugar previsto para la entrevista. Por ende, participaron en el estudio doce profesionales en total, distribuidos en las siete regiones sanitarias de la ciudad.

### Recolección de datos

La recolección de datos se realizó a través de una única entrevista semiestructurada, individual, presencial, grabada en soporte de audio, mediante grabadora digital, sin grabación de imágenes, con una duración promedio de cuarenta y cinco minutos.

Para ello, se utilizó un cuestionario semiestructurado con preguntas sobre conocimiento de los profesionales, estrategias y habilidades utilizadas, intenciones y metas, efectividad del manejo, rol del profesional, apoyo social y profesional, barreras, facilitadores y sentimientos. También se utilizó un instrumento para caracterizar a los participantes con edad, estado civil, tiempo de graduado, color/etnia, carga horaria, tiempo en el cargo e ingreso.

La recolección de datos fue realizada por dos investigadores, una estudiante de grado y un enfermero (estudiante de maestría), ambos previamente capacitados.

### Procedimiento para la recolección de datos

Para comenzar la recolección de datos, se contactó de forma telefónica el responsable de la unidad y se acordó un horario para presentar la investigación, de manera que no comprometiera la rutina y la atención que se le brinda a la población.

En esa visita, los investigadores responsables de la recolección de datos presentaron la propuesta de investigación y los procedimientos de recolección de datos a todos los enfermeros de la unidad.

Luego de este primer contacto con los enfermeros, se obtuvieron tres tipos de respuesta: 1) negativa a participar; 2) aceptación con programación de la entrevista; 3) aceptación y realización de la entrevista en el momento. En el primer caso, la unidad fue excluida de la lista y se realizó un nuevo sorteo en el grupo de la unidad visitada. Entre los motivos citados por los profesionales para negarse a participar están la carga de trabajo y la falta de colegas para reemplazar al profesional durante la entrevista.

En la segunda situación, la entrevista se programó en un día/lugar elegido por el participante, y se le informó que podía cancelar o reprogramar en cualquier momento. Cuando alguna situación le impedía a un participante realizar la entrevista, se programaba una nueva fecha, en un plazo de hasta siete días desde la fecha inicial.

En las unidades en las que más de un profesional por unidad manifestó interés en participar, los investigadores brindaron nuevas orientaciones sobre la metodología y el profesional fue incluido en la lista de reserva para cuando fuera imposible entrevistar al profesional seleccionado inicialmente. En la tercera situación, la entrevista se realizó en el momento en el que el profesional aceptó participar.

### Procesamiento y análisis de datos

Todos los audios de las entrevistas fueron transcritos íntegramente y las identificaciones de los participantes, personas e instituciones mencionadas fueron reemplazadas por códigos que eran dominio exclusivo de los investigadores. A cada entrevista realizada se le asignó un código que describía el orden de la entrevista realizada y el grupo al que pertenecía (ej.: G1:E1, G2:E2, G3:E3, etc.).

 El instrumento TDF ( Figura 1 ) proporcionó una estructura de los factores determinantes (dominios y constructos) del trabajo del enfermero en situaciones de crisis de salud mental, es decir, facilitó identificar los determinantes que establecen, mantienen y modifican la conducta ^(^ ^26^ ^)^ . 

 Para el proceso de análisis se utilizó el método deductivo, que comprende cinco fases: 1) lectura y exploración del material; 2) codificación y organización del contenido de los discursos en los constructos y dominios que mejor reflejen el contenido transcrito; 3) distribución absoluta y proporcional del número de discursos; 4) elaboración de temas considerando las intersecciones de los discursos y los marcos teóricos interpretativos; 5) elaboración de un mapa ilustrativo de las relaciones entre temas/dominios ^(^ ^26^ ^,^ ^29^ ^)^ . 

**Figura 1:** - *Theorical Domains Framework* : sus dominios, definición y constructos

**Dominio**	**Definición**	**Constructo**
1. Conocimiento	Conciencia de la existencia de algo.	-Conocimiento (incluido el conocimiento de la condición/base científica) -Conocimiento procedimental -Conocimiento sobre el contexto de la tarea
2.Capacidad	Habilidad o competencia adquirida a través de la práctica.	-Desarrollo de la capacidad -Competencia -Habilidad -Habilidades interpersonales -Práctica -Evaluación de las capacidades
3.Identidad y rol profesional/social	Conjunto de conductas coherentes y presencia de cualidades personales de un individuo en un entorno social o laboral.	-Identidad profesional -Rol profesional -Identidad social -Identidad -Límites profesionales -Confianza profesional -Identidad de grupo -Liderazgo -Compromiso organizacional
4.Creencia en las capacidades	Aceptación de la verdad, realidad o validez sobre una habilidad, talento o aptitud que una persona puede utilizar de manera constructiva.	-Autoconfianza -Competencia percibida -Autoeficacia -Control conductual percibido -Creencias -Autoestima -Empoderamiento -Confianza profesional
5.Optimismo	Confianza en que las cosas saldrán bien o que se alcanzarán los objetivos deseados.	-Optimismo -Pesimismo -Optimismo poco realista -Identidad
6.Creencia sobre las consecuencias	Aceptación de la verdad, realidad o validez de los resultados de una conducta en una situación determinada.	-Creencias -Expectativas de resultados -Características de las expectativas de resultados. -Arrepentimiento anticipado -Consecuentes
7.Refuerzo	Aumentar la probabilidad de una respuesta estableciendo una relación de dependencia o contingencia entre la respuesta y un estímulo determinado.	-Recompensas (proximales/distales, con valor/sin valor, probables/improbables) -Incentivos -Castigo -Consecuentes -Refuerzo -Contingencias -Sanciones
8.Intenciones	Decisión consciente de tener una conducta o la determinación de actuar de una determinada manera.	-Estabilidad de intenciones -Modelo de etapas de cambio -Modelo transteórico y etapa de cambio
9.Metas	Representaciones mentales de resultados o estados finales que un individuo desea lograr.	-Metas (distal/proximal) -Prioridad de las metas -Meta/Establecimiento de metas -Metas (autónomas/controladas) -Plan de acción -Intención de implementación
10.Procesos de memoria, atención y decisión	Habilidad para retener información, centrarse selectivamente en aspectos del entorno y elegir entre dos o más alternativas.	-Memoria -Atención -Control de la atención -Toma de decisiones -Sobrecarga cognitiva/cansancio
11.Contexto ambiental y recursos	Cualquier circunstancia de la situación o entorno de una persona que desalienta o fomenta el desarrollo de capacidades y habilidades, la independencia, la competencia social y el comportamiento adaptativo.	-Estresores ambientales -Recursos materiales -Cultura/clima organizacional -Eventos destacados/incidentes críticos -Interacción persona vs. entorno -Barreras y facilitadores
12.Influencias sociales	Procesos interpersonales que pueden hacer que los individuos cambien sus pensamientos, sentimientos o comportamiento.	-Presión social -Normas sociales -Conformidad del grupo -Comparaciones sociales -Normas del grupo -Apoyo social -Poder -Conflicto entre grupos -Alienación -Identidad de grupo -Modelado
13.Emoción	Patrón complejo de reacción, que involucra elementos de la experiencia, conductuales y fisiológicos, mediante el cual un individuo intenta lidiar con un tema o evento personalmente significativo.	-Miedo -Ansiedad -Afecto -Estrés -Depresión -Afecto positivo/negativo -Burnout
14.Regulación de la conducta	Cualquier cosa destinada a gestionar o cambiar acciones observadas o medidas objetivamente.	-Autocontrol -Cambiar hábitos -Plan de acción

Fuente: Extraído de la guía de uso del *Theorical Domains Framework* ^(^ ^26^ ^)^

 Este proceso fue realizado de forma independiente, por dos investigadores capacitados en el uso del instrumento y, posteriormente, los análisis fueron comparados y discutidos hasta llegar a un consenso. Cuando no hubo consenso, el discurso/extracto de la entrevista fue asignado a todos los dominios enumerados por los investigadores ^(^ ^26^ ^)^ . 

 Todo el análisis fue guiado por la elaboración de tablas que contienen los discursos/extractos de los participantes. La interpretación de los enunciados se realizó basándose en las definiciones de los constructos descritos en el TDF y comparándolos con los marcos teóricos del AC y MHD. Los datos de caracterización de la muestra y distribución cuantitativa (absoluta y proporcional) se organizaron y analizaron en un documento *Excel* ( *Microsoft* ). 

### Aspectos éticos

El estudio fue aprobado por el Comité de Ética en Investigaciones con Seres Humanos, según CAAE: 59722622.9.0000.0021.

## Resultados

Participaron del estudio doce profesionales, siete eran del sexo masculino (58,3%), con una edad promedio de 33 años (máximo 47, mediana 31,5 y mínimo 24), la mayoría era soltero (58,3%), ocho (66,7%) se consideraron morenos, dos (16,7%) blancos y uno negro (8,3%). En cuanto al tiempo en el cargo, osciló entre uno y diez años, con un promedio de tres años y medio. El ingreso familiar promedio de los profesionales era de R$ 8.550 reales.

 A partir del análisis se identificaron constructos de los 14 dominios, con la siguiente distribución ( Tabla 1 ). 

 A partir del análisis y la interpretación, los datos se agruparon en dos temas, a saber “El trabajo del enfermero: determinantes materiales y sociales (TEMA-01)” y “Condiciones subjetivas del enfermero en situaciones de crisis de salud mental (TEMA-02)” (ver Figura 2 ). 

**Tabla 1:** - Distribución absoluta y proporcional (por dominio y con respecto al total) de constructos y dominios. Campo Grande, MS, Brasil, 2022-2023

**Número de eventos**	Proporción por dominio (% ^*^ )	**Constructo**	**Dominio**
61	14,48	Conocimiento (incluido el conocimiento de la condición/base científica)	Conocimiento
74		Conocimiento procedimental	
97		Conocimiento sobre el contexto de la tarefa	
44	17,01	Recursos/recursos materiales	Contexto ambientaly recursos
3		Estresores ambientales	
15		Cultura/clima organizacional	
96		Barreras y facilitadores	
10		Eventos destacados/incidentes críticos	
98		Interacción persona vs. entorno	
9	14,61	Capacidad	Capacidades
15		Desarrollo de la capacidad	
59		Competencia	
20		Capacidades interpersonales	
31		Práctica	
55		Evaluación de las capacidades	
42		Habilidades	
49	9,55	Identidad Profesional	Identidad y rol profesional/social
65		Rol profesional	
3		Liderazgo	
34		Identidad de grupo	
10	8,16	Autoconfianza	Creencia en las capacidades
5		Competencia percibida	
6		Autoeficacia	
43		Creencias	
3		Empoderamiento	
44		Control Conductual Percibido	
18		Confianza profesional	
23	7,21	Creencias	Creencia sobre las consecuencias
25		Expectativa de Resultados	
15		Características de las expectativas de resultados	
9		Arrepentimiento anticipado	
42		Consecuentes	
1	1,20	Identidad	Optimismo
7		Optimismo	
9		Pesimismo	
2		Optimismo irrealista	
17	14,17	Recompensa (proximal/distal, valido o no válido, probable o improbable)	Refuerzo
1		Incentivos	
49		Castigo	
43		Consecuentes	
21		Refuerzo	
92		Contingencias	
1		Sanciones	
8	0,51	Estabilidad de Intenciones	Intenciones
17	3,92	Metas (distales/proximales)	Metas
5		Metas (autónomas/controladas)	
13		Meta/Establecimiento de metas	
13		Prioridad de las metas	
12		Intenciones de implementación	
2		Plan de acción	
4	2,97	Presión social	Influencias sociales
1		Comparaciones sociales	
1		Conformidad del grupo	
1		Normas sociales	
37		Apoyo social	
2		Conflicto entre grupos	
1		Poder	
2	3,35	Miedo	Emociones
1		Ansiedad	
27		Afecto	
0		Estrés	
17		Afecto positivo/negativo	
6		Agotamiento	
13	0,82	Autocontrol	Regulación de la conducta
1		Cambiar hábitos	
18		Plan de acción	
1	2,02	Memoria	Memoria, atención y proceso de decisión
11		Control de la atención	
8		Atención	
11		Toma de decisiones	
1		Sobrecarga cognitiva/cansancio	

^*^ Valor en porcentaje de la distribución proporcional (relativa) de cada dominio

**Figura 2:** - Mapa de los temas y las intersecciones entre los dominios del TDF*

* *Theorical Domains Framework*

Nota: Línea verde: relación única con el Tema 01; línea azul: Relación única con el Tema 02; línea roja: relación entre los dos temas; línea discontinua: relación entre constructos; elaboración propia, basado en la literatura ^(^ ^29^ ^)^

### El trabajo del enfermero: determinantes materiales y sociales

Se mencionaron actividades relacionadas con el trabajo asistencial del enfermero en los dominios capacidad y conocimiento procedimental, surgieron prácticas como: derivación de usuarios al Servicio de Urgencia y Emergencia (SUE) o a servicios especializados como el Centro de Atención Primaria de Salud (CAPS), Centros de Apoyo a la Salud de la Familia (CASF); internación; clasificación de riesgo; contención farmacológica y/o mecánica; recepción, visitas domiciliarias en casos excepcionales y búsqueda activa.

*(...) dos alternativas, una es llamar a urgencias y emergencias para poder contenerlo, para que no se automutile […].* (E1) 

*(...)ingresó aquí a la unidad y pasa por un proceso de escucha, es obvio que si está en un momento de gran crisis [...] se trata como una emergencia, la máxima clasificación aquí. [...] será evaluado y enviado a enfermería para realizar una clasificación de riesgo [...] derivamos pacientes a través del sistema de vacantes [...] (E08)*

*(...) lo derivamos al CAPS, porque allí lo van a evaluar, luego lo envío a consulta con un psicólogo, a veces con un psiquiatra y, si es necesario, a internación.* (E10) 

*Recepción. Las personas necesitan atención y afecto, entender que hay alguien que se preocupa por ellas, sin juzgar. Entonces, creo que este es el punto clave de la Atención Primaria [...] recibir al usuario considerando su individualidad.* (E09) 

El enfermero se describe (identidad y rol profesional/social) como el profesional que tiene mayor contacto con los usuarios, responsable de la recepción, apoyo y diálogo y de la clasificación/organización de la atención. Además, mencionaron la responsabilidad de articular el equipo, la familia y los componentes de la RAPS.

*Enfermería es el primer contacto cuando estoy hablando con el paciente [...] pero tenemos más, digamos, familiaridad incluso con las familias [...] Y para dar apoyo emocional también, tenemos la función de encargarnos de la parte emocional del paciente, brindarle apoyo.* (E02) 

*Si el paciente no está estable, aquí tratamos de estabilizarlo, pasa por la consulta de enfermería, se realiza la clasificación y luego se lo deriva al médico que estabiliza el cuadro con algún fármaco [...].* (E08) 

*Mire, yo veo al enfermero como una persona que va a organizar la atención, a dirigir bien la atención [...].* (E10) 

*Nosotros hacemos un relevamiento de la demanda y es el enfermero quien coordina este proceso, incluso con la familia, y somos nosotros quienes estaremos en la casa, para orientarlos y poder llevar los casos al equipo multidisciplinario para discutirlos.* (E03) 

*Nosotros también tenemos un rol, cómo lo puedo decir, para tratar de resolver también estos casos. [...] ...el cuadro que tiene el paciente.* (E05) 

Las acciones y prácticas que se realizan en la APS, según los profesionales, tienen consecuencias para la adherencia al tratamiento (mejora/empeora); dificultades para monitorear e identificar los casos; ruptura de vínculos y recurrencia de crisis.

*Y otra cosa es la adherencia. Adherencia al tratamiento. Entonces, el paciente piensa que está bien y luego ya no cumple con el tratamiento y luego vuelve la crisis. [...].* (E03) 

*Entonces cuando utilizamos este método de asistencia que es recibir, escuchar, terminamos teniendo una alta adherencia por parte de los pacientes [...]. Cuando puede comprender, entender el proceso de salud y enfermedad, eso le facilita la adherencia al tratamiento [...] le ayuda a cambiar su estilo de vida [...].* (E05) 

*(...) entonces, el paciente no crea un vínculo con el médico del equipo, no crea un vínculo con el enfermero que representa a todo el equipo. [...] entonces ¿cómo va a crear un vínculo el paciente si cada vez que viene aquí lo atiende un médico diferente? [...].* (E08) 

En lo que respecta al contexto ambiental y los recursos, los enfermeros informan: escaso número de vacantes, dificultades para acceder a los servicios (largos tiempos de espera, por ejemplo), desguace de estructuras y espacios de atención adecuados, falta de recursos humanos y alta rotación. Este contexto se relacionó con consecuencias como sobrecarga profesional y reducción del número de profesionales que brindan atención al usuario.

 (...) *hay mucho desgaste, en las cuarenta horas del profesional, hay mucho sufrimiento por eso en ese proceso con toda esa carga [...].* (E09) 

*(...) Y mi preocupación también se refiere a veces a la reducción del limitado número de vacantes disponibles para el tratamiento oportuno de estos pacientes.* (E02) 

*(...) luego la persona entra en una lista, espera de 5 a 6 meses para recibir atención [...].* (E01) 

*(...) la sobrecarga del Sistema Único de Salud (SUS), la sobrecarga del propio sistema y la falta de profesionales, es un desguace del SUS lo que está ocurriendo en el país [...].* (E04) 

*A veces la estructura física no tiene suficiente espacio, hay una habitación para cuatro enfermeros, por lo que muchas veces no hay dónde llevar al paciente para hablar mejor con él.* (E12) 

En cuanto a las influencias sociales (apoyo social, presión social, conflicto entre grupos), se describió que había apoyo para realizar la derivación a los servicios especializados (CAPS y CASF) y Servicios de Urgencia y Emergencia (SUE), pero que estaban comprometidas la comunicación y la contraderivación. También se citaron interferencias del equipo médico en la autonomía de los enfermeros.

*(...) no hay comunicación, no hay contraderivación, no hay una cierta autonomía de que voy a derivar al paciente y tener acceso a todo lo que se está haciendo […]. Entonces es un bucle infinito porque tenemos que estar todo el tiempo atrás de la información, no la tenemos [...]. (E08)*

*(...) Y puedo llamar al bombero, puedo llamar al Servicio de Atención Móvil de Emergencias (SAMU) también [...]. (E02)*

*(...) porque no tengo acceso, o mi categoría no me lo permite, o lo que puedo hacer, a veces, lo deshace otro profesional, por motivos médicos [...].* (E12) 

Las actividades mensuales de educación/discusión también se mencionaron como una forma de apoyo social.

*Todos los meses tenemos reuniones mensuales que se llevan a cabo en el Centro Especializado Municipal (CEM) con el equipo de salud mental donde se abordan diversos temas relacionados con el flujo de pacientes y demandas regulatorias. Discutimos casos, presentamos casos reales que suceden en las unidades para discutir y ver qué podríamos haber hecho, enumeramos las potencialidades y debilidades de este servicio.* (E05) 

### Condiciones subjetivas del enfermero en situaciones de crisis de salud mental

Los enfermeros reportaron inseguridad/falta de capacidad técnica, además de situaciones donde se expresó estigma hacia los usuarios.

*(...) la falta de capacidad técnica [...] Hago lo mejor que puedo como enfermera, todo en base a todo lo que aprendí, pero ¿soy experta en esto? ¡No! ¿Soy parte de un grupo que siempre tiene educación continua sobre esto para saber manejar los casos? ¡No!* (E08) 

*(...) sí, hay algunos prejuicios de orden religioso, de orden social, de orden económico [...] Creo que uno de los factores es la discriminación incluso con paciente con problemas mentales. Muchas veces dicen que es posesión demoniaca [...] dicen que el paciente es “vago”.* (E12) 

La crisis de salud mental (conocimiento de la condición) se asoció con la noción de urgencia/emergencia psiquiátrica y, en ocasiones, se utilizó el término “brote”. Además, los participantes, al especificar los tipos de crisis, citaron signos/síntomas de trastornos mentales.

*La crisis es el brote psiquiátrico, considero que es una situación en la que el individuo está fuera de sí, hablando en términos de urgencia [...].* (E02) 

*Puede ser desde un ataque de ansiedad, un ataque de pánico, pacientes con un brote por algún tipo de trastorno, trastorno bipolar, pacientes esquizofrénicos con algún tipo de brote [...].* (E06) 

Como excepción, se destacan dos informes que describen una comprensión de la crisis más cercana al modelo psicosocial.

*Entonces entiendo la crisis de salud mental como un proceso [...], pero algo inesperado con lo que el paciente por sí solo no es capaz lidiar en ese momento desafiante o incluso algún evento externo [...].* (E04) 

*(...) Creo que es todo problema que hace que la persona ya no puede tener buen discernimiento, psíquico y mental, que termina afectando las actividades cotidianas, tanto las relaciones familiares como las relaciones con los amigos y el trabajo. Llega al punto en que afecta el proceso de vinculación social de la persona... [...]* (E11) 

En cuanto a las creencias (capacidades y consecuencias), los participantes dijeron que creen que reducen los riesgos, pero que su trabajo no contribuye al tratamiento de los trastornos, sino que sólo controla puntualmente las crisis.

*(...) no previene un problema mayor, pero logramos minimizar los riesgos de la gravedad de la enfermedad o de un brote en sí o de un intento de suicidio [...] una reducción del daño [...]. (E02)*

*(...) la causa del problema no se resolvió, ese evento desencadenante, a veces, ni siquiera se resolvió [...] creo que nuestra atención hoy está haciendo al paciente adicto a la medicación, no contribuye a controlar la enfermedad bajo ninguna circunstancia.* (E06) 

Destacan que buscan con su trabajo (metas): reducción del sufrimiento, adecuación y regularización del tratamiento farmacológico, vinculación a servicios especializados y estabilización de los usuarios para que puedan reinsertarse en la sociedad.

*Entonces, el objetivo más grande es reducir el sufrimiento psicológico del paciente, porque no tiene sentido tener una lista vacía, pero tengo usuarios que sufren un dolor psicológico absurdo, les duele más que el dolor físico* (E09) 

*De hecho, cuando utilizamos estas estrategias intentamos que los pacientes sigan un tratamiento regular [...].* (E07) 

*Cuando se trata de la persona que sufre depresión, evitar que llegue a la etapa de autolesión, de automutilación, de autoexterminio [...]. ...hay que derivarlo al CAPS, al Centro de Apoyo Psicosocial que tenemos en la ciudad.* (E11) 

*Intentar estabilizarlos para que puedan vivir en la sociedad normal [...] ...intentar que pueda volver a tener vida social [...]* (E03) 

Por último, los enfermeros informaron que sienten angustia, miedo, inquietud, falta de preparación/incapacidad, frustración, culpa y cansancio.

*La frustración creo que es la más fuerte. [...]. De ahí viene la frustración de no poder hacer más de lo que hacemos para ayudar.* (E11) 

*Entonces esto le genera mucha angustia al profesional [...].* (E09) 

*Hay muchos profesionales que no tienen capacidad, tienen miedo, no saben cómo actuar, muchas veces en situaciones de crisis [...].* (E12) 

*Pero yo no me siento así, no siento placer al atender este tipo de demandas, ¿sabes? No es algo que me llame mucho la atención* . (E05) 

## Discusión

Los resultados refuerzan la tesis de que el trabajo del enfermero se restringe al ámbito “técnico” centrado en diagnósticos psiquiátricos, permeado por contradicciones objetivas y subjetivas que interfieren directa o indirectamente en las estrategias que utilizan en su proceso de trabajo y en la atención de personas en situación de crisis de salud mental.

 De hecho, incluso con herramientas y tecnologías para que los enfermeros realicen un trabajo que promueva una experiencia positiva de la crisis, las condiciones y los recursos disponibles terminan incentivando una ejecución protocolizada y exhaustiva, debido a la sobrecarga del profesional, de etapas fijas de atención, clasificación de riesgos, identificación de signos/síntomas y derivación de usuarios a servicios especializados, que, directa o indirectamente, promueven el fenómeno de medicalización y patologización de los problemas de salud mental ^(^ ^12^ ^,^ ^16^ ^,^ ^30^ ^)^ . 

 Además, las condiciones de trabajo aumentan las dificultades para organizar estrategias de educación continua, esenciales para desarrollar habilidades adecuadas para enfrentar las condiciones de salud; situación que se identificó tanto en el presente trabajo como en la literatura nacional e internacional ^(^ ^16^ ^,^ ^30^ ^)^ . 

 Por lo tanto, se puede suponer que persiste en la APS una perspectiva manicomial, basada en un proyecto neoliberal de asfixia político-económica, guiado por la austeridad, que en un país como Brasil, se manifiesta en la reducción paulatina del Estado y de su capacidad para implementar políticas de salud pública ^(^ ^5^ ^,^ ^21^ ^-^ ^24^ ^)^ . 

 Para el MHD, este proyecto es fundamental para transformar la salud mental y la crisis en una mercancía. Para lograrlo, es necesario “simplificar” la experiencia del sufrimiento humano y encuadrarla en criterios diagnósticos preestablecidos, en un procedimiento que facilite dirigir las demandas a la industria farmacéutica, a las redes de salud privadas y/o religiosas; a las comunidades terapéuticas ^(^ ^25^ ^,^ ^31^ ^-^ ^34^ ^)^ . 

 Además, también es necesario que el enfermero de la APS, junto con el usuario y sus compañeros, viva en un ambiente precario, marcado por sobrecarga, baja resolución, oferta limitada de servicios, alta rotación, dificultad para construir vínculos, inestabilidad de los vínculos de trabajo y con el incentivo de la flexibilización/tercerización ^(^ ^21^ ^-^ ^23^ ^,^ ^30^ ^-^ ^32^ ^)^ . 

 De esta manera, el trabajo del enfermero, esencialmente concreto y dotado de valor de uso intrínseco, acaba alienándose de su función social, que es la producción de salud para el cuidado, y se constituye únicamente como una fuerza de trabajo que realiza actividades estandarizadas e, indirectamente, pasa a formar parte de la cadena de valorización del capital (valor que genera valor), agilizando la circulación y el intercambio de bienes ^(^ ^25^ ^,^ ^33^ ^)^ . 

 Por otro lado, como se puede ver en el estudio, a veces la comprensión de la salud mental se vuelve indistinguible de los diagnósticos psiquiátricos y actúa sólo como su negativo (“no tener un trastorno”), y la crisis es vista como la situación aguda grave de un trastorno mental; fenómeno que está presente también en la literatura ^(^ ^30^ ^-^ ^33^ ^)^ . 

 Es la socialización de un fetiche técnico, es decir, lo subjetivo adquiere carácter objetivo, es simplificado, identificado fácilmente, categorizado y controlado; el sufrimiento adquiere una forma más propicia para la dinámica del mercado. La psiquiatría, de esta manera, adquiere poder social y coercitivo, descontextualiza las conductas del entorno (actual y anterior) del individuo, de las variables de refuerzo positivo y negativo y de la historia personal de aprendizaje de cada individuo ^(^ ^14^ ^,^ ^25^ ^,^ ^31^ ^-^ ^32^ ^)^ . 

 En otras palabras, los comportamientos visibles (públicos) y las emociones (privadas) son explicados por los trastornos mentales y toda la historia de vida -violencia, abandono, maltrato, etc.- se trata, como máximo, como “factores de riesgo individuales no modificables”. Esta forma de “explicación” es diariamente validada y compartida por los aparatos ideológicos del capital, dado que es necesario gestionar constantemente los productos (el sufrimiento) de las contradicciones de las relaciones de explotación laboral. Por lo tanto, el capital se beneficia del sufrimiento que él mismo genera y, es más, individualiza los determinantes de este sufrimiento, culpando a los individuos por los males sociales del modo de producción capitalista ^(^ ^14^ ^,^ ^25^ ^,^ ^31^ ^-^ ^32^ ^)^ . 

 La comprensión de la salud mental y de las condiciones precarias que enfrentan los profesionales también puede explicar por qué acciones que teóricamente estarían asociadas a la atención psicosocial, como la recepción y el vínculo, se convierten en etapas de un proceso “técnico”, desprovisto de su carácter humanizado que requiere “la dedicación de un tiempo distinto del que llevan las demandas convencionales” ^(^ ^11^ ^,^ ^15^ ^,^ ^18^ ^,^ ^31^ ^)^ . 

 Cabe destacar, que este panorama no es exclusivo de los países periféricos, como Brasil, sino que está presente en todos los países, en menor o mayor medida. Por ejemplo, en los Estados Unidos, la falta de un sistema de salud pública y el crecimiento de las desigualdades sociales han afectado desproporcionadamente a la población más vulnerable, hay un alarmante crecimiento de la población sin hogar, del encarcelamiento masivo y empeoramiento de las condiciones de salud ^(^ ^5^ ^)^ . 

 En China, a pesar de los esfuerzos por reducir la inequidad en el acceso a la atención médica, la disminución del número de trabajadores de salud mental, incluso de enfermeros, y las dificultades para capacitar a estos profesionales han planteado desafíos para la organización de un sistema comunitario de salud mental. Una situación similar se puede encontrar en otros países de Europa y Oriente Medio ^(^ ^6^ ^,^ ^10^ ^,^ ^35^ ^-^ ^37^ ^)^

 Además, se destaca que, tanto en Brasil como en otros países, los profesionales de enfermería deben lidiar con las inequidades de los marcadores sociales de género y raza; la mayor parte de la fuerza laboral de enfermería está compuesta por mujeres, y en Brasil, por mujeres negras que enfrentan jornadas de trabajo dobles o triples ^(^ ^5^ ^-^ ^6^ ^)^ . 

 No sorprenden las repercusiones emocionales y afectivas (frustración, miedo, etc.) que mencionaron los enfermeros. Ante posibilidades y acciones limitadas, escaso apoyo social, falta de educación continua, poca resolución y condiciones de vida cada vez más precarias, el sentimiento de frustración del profesional es justificable, dado que las emociones son reacciones al entorno (comportamiento reflejo aprendido) ^(^ ^14^ ^)^ . 

 Por ende, la persistencia de un profesional en construir o no nuevas estrategias, probar nuevas posibilidades, buscar nuevas redes de comunicación, entre otras, se relaciona con un patrón específico de refuerzo en la historia del profesional, es decir, el motivo y los intervalos que llevaron al profesional a probar alternativas y si tuvo éxito o no. Por ejemplo, la cantidad de veces que un profesional intentó crear un proyecto terapéutico único con el CASF por la cantidad de éxitos obtenidos ^(^ ^14^ ^)^ . 

 Además, las repercusiones emocionales/psicológicas también se pueden encontrar en otros países. Los enfermeros se enfrentan a situaciones de violencia, acoso moral, *bullying* por parte de sus compañeros de trabajo y problemas organizativos que minan la capacidad de resolución de la atención. La literatura internacional ha revelado un escenario complejo de enfermedad en el trabajo que en ocasiones conduce a desenlaces como el suicidio ^(^ ^37^ ^)^ . 

Por lo tanto, es problemático responsabilizar sólo al enfermero por los resultados en la atención a las crisis de salud mental, ya que no existen condiciones concretas para crear acciones relacionadas con el modelo psicosocial.

 Por ello, individualizar el problema sólo aumentaría el número de situaciones que pueden reforzar emociones “negativas”; frustración/culpa. A eso se le suma las falencias en la estructuración sistemática de acciones de educación continua, la sobrecarga, la baja disponibilidad de tiempo y recursos inadecuados que hacen improbable la aplicación sistemática de las mejores estrategias de atención en situaciones de crisis de salud mental ^(^ ^14^ ^,^ ^16^ ^,^ ^30^ ^-^ ^31^ ^)^ . 

 No hay duda, y la literatura refuerza este punto de manera exhaustiva, que es necesario invertir en la formación y educación continua de los enfermeros para que afronten situaciones de crisis. Sin embargo, para que las inversiones sean posibles y haya condiciones concretas favorables para el desarrollo de la atención psicosocial por parte del enfermero, el Estado y su población deben superar la política neoliberal, prohibiendo completamente la lógica de la mercancía en el campo de la salud ^(^ ^5^ ^,^ ^16^ ^,^ ^31^ ^-^ ^32^ ^,^ ^37^ ^)^ . 

Como categoría profesional con fuerte poder social, que convive y tiene contacto diario con su comunidad, el enfermero puede contribuir a esta lucha, fortaleciendo su protagonismo político-social con mayor inserción en la lucha de la clase trabajadora. Por lo tanto, debe actuar activamente a través de sindicatos, consejos, partidos de vanguardia, movimientos e iniciativas sociales, luchando por condiciones laborales justas y la superación de la pobreza, la falta de vivienda, saneamiento, trabajo digno, entre otros problemas que aquejan a la sociedad capitalista.

En cuanto a las limitaciones del estudio, se destaca que la APS es sólo uno de los escenarios de la RAPS donde los enfermeros pueden brindar atención a personas en situaciones de crisis de salud mental. Por ende, es vital que se realicen nuevos estudios para poder analizar todo el contexto de la RAPS, en un proceso que involucre diferentes categorías de profesionales de la salud y otros actores, incluidos los usuarios.

## Conclusión

En síntesis, el estudio permitió analizar cómo se estructura el trabajo del enfermero en la APS ante situaciones de crisis de salud mental de los usuarios, y describe sus contradicciones objetivas y subjetivas, una comprensión mayoritariamente manicomial y un trabajo delimitado por el fenómeno de alienación y fetichización de la “técnica” que sirve para insertar indirectamente el trabajo en salud concreto en la cadena de valorización del capital.

El enfermero es consciente de que la atención que brinda en situaciones de crisis puede realizarse de otra manera, pero, dada la sobreexplotación y precariedad de los recursos, hay pocas condiciones para ello e implica para los profesionales repercusiones emocionales/afectivas de frustración, miedo, entre otras.

Por último, el uso de marcos teóricos críticos y de la sistematización metodológica que permite el instrumento TDF, es fundamental en esta situación, dado que revela que es necesario que haya una mayor comprensión teórico-científica de las limitaciones sociales y materiales del trabajo en salud.

# Translation: Situações de crise de saúde mental: o trabalho do enfermeiro na Atenção Primária à Saúde ^*^

**DOI:** 10.1590/1518-8345.7015.4357

**Authors:** Gessner Bravo de Paula, Nur Mohamad Ali El Akra, Lucas Ferraz Córdova, Lucilene Cardoso, Ana Carolina Guidorizzi Zanetti, Bianca Cristina Ciccone Giacon Arruda

**Affiliations:** ^1^ Universidade Federal do Mato Grosso do Sul, Instituto Integrado de Saúde, Campo Grande, MS, Brasil.; ^2^ Secretaria do Estado de Saúde, Coordenadoria Estadual do Telessaúde, Campo Grande, MS, Brasil.; ^3^ Bolsista da Fundação de Apoio ao Desenvolvimento do Ensino, Ciência e Tecnologia do Estado do Mato Grosso do Sul (FUNDECT), Brasil.; ^4^ Bolsista da Coordenação de Aperfeiçoamento de Pessoal de Nível Superior (CAPES), Brasil.; ^5^ Universidade Federal do Mato Grosso do Sul, Faculdade de Ciências Humanas, Campo Grande, MS, Brasil.; ^6^ Universidade de São Paulo, Escola de Enfermagem de Ribeirão Preto, Centro Colaborador da OPAS/OMS para o Desenvolvimento da Pesquisa em Enfermagem, Ribeirão Preto, SP, Brasil.

**Keywords:** Atenção Primária à Saúde, Enfermeiras e Enfermeiros, Intervenção na Crise, Modelos de Atenção, Saúde Mental, Trabalho

## Abstract

**Objetivo:**

analisar o trabalho do enfermeiro na Atenção Primária à Saúde diante de situações de crise de saúde mental.

**Método:**

estudo descritivo-exploratório, com abordagem qualitativa, subsidiada pelos referenciais teóricos-interpretativos da análise do comportamento e do materialismo histórico-dialético. Os dados foram coletados por meio de uma entrevista semiestruturada com doze enfermeiros da Atenção Primária à Saúde e analisados pela técnica dedutiva proposta pelo *Theorical Domains Framework* , referencial metodológico adotado.

**Resultados:**

os dados analisados possibilitaram a elaboração de dois temas: “O trabalho do enfermeiro: determinantes materiais e sociais” e “Condições subjetivas do enfermeiro diante de situações de crise de saúde mental”. O trabalho do enfermeiro foi pautado pela execução protocolar de etapas “técnicas” relacionadas à clínica psiquiátrica, com compreensão da crise como “surto psiquiátrico”.

**Conclusão:**

o estudo possibilitou analisar o trabalho do enfermeiro diante das situações de crise, descrevendo as contradições objetivas e subjetivas, sua compreensão do fenômeno da crise a as repercussões emocionais desse trabalho nos profissionais. Evidenciou-se um trabalho atravessado pela alienação e pelo *fetiche* da técnica, situação que levanta a necessidade de atuação, organização e mobilização político-social da categoria de enfermagem em torno da luta contra o modelo de atenção manicomial e consolidação da perspectiva da Reforma Psiquiátrica.

## Destaques

**(1)** Atuação manicomial protocolar de identificação e redução de sintomas da crise.

**(2)** Redução da experiência da crise como fenômeno psicopatológico.

**(3)** Utilização de referenciais teórico-críticos alinhados às propostas da atenção psicossocial.

**(4)** Referencial metodológico-analítico validado internacionalmente.

**(5)** Necessidade de atuação político-social por parte da enfermagem.

## Introdução

 O comprometimento da saúde mental vem apresentando um crescimento expressivo em nível mundial entre a população geral. Em 2019, estimava-se que, aproximadamente, 13% da população mundial (970 milhões de pessoas) viviam com algum tipo de comprometimento na saúde mental, sendo os mais prevalentes os transtornos de ansiedade (31%), depressão (28,9%) e aqueles decorrentes do uso de álcool e outras drogas ^(^ ^1^ ^)^ . 

 A saúde mental é definida pela Organização Mundial de Saúde como um estado no qual o indivíduo consegue funcionar, prosperar, lidar com as dificuldades, conectar-se e contribuir para a melhoria de sua comunidade. Por conseguinte, são problemas intimamente relacionados/determinados pelas condições físicas, biológicas, socioculturais e comportamentais ^(^ ^2^ ^-^ ^4^ ^)^ . 

 Ademais, a literatura vem destacando como o ambiente político influencia esses determinantes de saúde. Nesse sentido, evidências indicam que a difusão mundial da política de austeridade econômica neoliberal está aumentando drasticamente as taxas de adoecimento mental, diminuindo a expectativa de vida e elevando iniquidades no acesso aos serviços de saúde ^(^ ^3^ ^-^ ^5^ ^)^ . 

 Em países da periferia do capitalismo, como o Brasil, essa política vem se configurando pela rápida e gradual destruição das capacidades de organização/coordenação dos sistemas de saúde, precarização das condições de trabalho e da qualidade do cuidado. Nos países centrais do capitalismo, a política neoliberal incorporou a lógica da eliminação de restrições ao mercado, com a rápida diminuição, ou mesmo extinção, dos mecanismos de assistência pública à saúde ^(^ ^3^ ^-^ ^10^ ^)^ . 

 Além disso, a política neoliberal levou a uma desarticulação organizativa e do poder de “barganha” dos trabalhadores, com crescimento alarmante da informalidade e dos planos privados de saúde com alto custo e pouca efetividade ^(^ ^4^ ^)^ . É diante desse contexto sociopolítico e epidemiológico que o enfermeiro constrói seu cuidado para usuários em situações de crise, expressão mais marcante dos problemas de saúde mental ^(^ ^1^ ^,^ ^4^ ^-^ ^11^ ^)^ . 

 A situação de crise em saúde mental pode ser compreendida de diferentes maneiras. No modelo manicomial, ela é definida como uma situação ou condição aguda sempre decorrente de um transtorno mental, associado à recusa do indivíduo por intervenções e/ou contato, impossibilidade pessoal de enfrentamento, com necessidade de intervenção imediata para diminuição dos sintomas ^(^ ^12^ ^-^ ^13^ ^)^ . 

 Por outro lado, o modelo psicossocial define a crise como um produto social com repercussões psicológicas, associado à capacidade dos sujeitos em responder a situações insuportáveis. Tem relação com o repertório psicossocial do indivíduo, resultado de uma interação complexa entre história individual, comportamento do indivíduo e o ambiente social/cultural ^(^ ^11^ ^,^ ^14^ ^-^ ^15^ ^)^ . 

 O modelo psicossocial de cuidado se caracteriza como uma forma específica de combinar técnicas, tecnologias e arranjos organizativos para atender aos problemas de saúde, buscando superar o modelo manicomial por meio de práticas comunitárias em meio aberto e para sujeitos ativos. Neste modelo, o enfermeiro tem a seu dispor diversas ferramentas para desenvolver seu trabalho e promover o cuidado nas situações de crise de saúde mental ^(^ ^16^ ^-^ ^17^ ^)^ . 

 Dentre as ações que esse profissional pode desenvolver, destacam-se: utilizar o acolhimento; desenvolver vínculo por meio de habilidades de comunicação/escuta; realizar avaliação da saúde física; identificar condições sociais e comportamentais de risco; criar planos de cuidados conjuntos; participar de ações de psicoeducação; conduzir e coordenar grupos terapêuticos; construir ações de educação permanente; promover a vinculação e acompanhamento dos usuários aos pontos adequados da rede. Além disso, pode orientar sobre o uso correto, benefícios, efeitos colaterais, duração e importância da adesão ao tratamento farmacológico ^(^ ^12^ ^-^ ^13^ ^,^ ^15^ ^,^ ^18^ ^)^ . 

 Para que essas ferramentas possam ser utilizadas, a literatura destaca a necessidade de que o cuidado de saúde mental de qualidade seja oferecido pela Atenção Primária à Saúde (APS); estratégia mundialmente reconhecida por aumentar o acesso, diminuir lacunas do cuidado e promover assistência integral, longitudinal e efetiva, e que esta esteja integrada a uma rede de serviços ^(^ ^19^ ^)^ . 

 Com esse objetivo, foi criada no Brasil a Rede de Atenção Psicossocial (RAPS), um arranjo organizativo de ações e serviços de saúde mental norteado pelo modelo de atenção psicossocial. Apesar das dificuldades, a RAPS promoveu o crescimento do investimento e das taxas de cobertura da população. Contudo, ainda hoje cerca de 77% da população brasileira ainda habita em regiões com baixa ou inexistente assistência da RAPS ^(^ ^20^ ^-^ ^22^ ^)^ . 

 Além disso, as condições materiais precarizadas de trabalho do enfermeiro, a baixa articulação e integração dos dispositivos da RAPS, dentre ele a APS, vêm se relacionando uma produção de cuidado de pouca resolutividade e linhas de cuidado incapazes de contemplar as necessidades de saúde mental em sua integralidade ^(^ ^19^ ^,^ ^21^ ^-^ ^24^ ^)^ . 

Deste modo, torna-se importante conhecer como está estruturado o trabalho de assistência do enfermeiro ao usuário em situações de crise de saúde mental, ou seja, quais são as condições objetivas/materiais e subjetivas presentes no cotidiano do enfermeiro.

Diante do exposto, questiona-se: O trabalho realizado por enfermeiro diante de indivíduos em situação de crise em saúde mental vem sendo desenvolvido para contemplar todas as possibilidades de atuação que ele possui na APS? Esta pesquisa parte da tese de que não, propondo que o trabalho do profissional está restrito a um fazer prático-técnico focalizado em categorias diagnósticas psiquiátricas, portanto, sendo realizado alienadamente e de modo fetichizado.

 Para isso, os resultados desse estudo foram interpretados pelo materialismo histórico-dialético (MHD) e pela análise do comportamento (AC). Para o MHD, o trabalho do enfermeiro consiste em trabalho concreto, aquele realizado e construído historicamente para atender às necessidades de saúde, produzindo assim valores de uso. Já para a AC, o trabalho do profissional é visto como comportamento operante (apreendido) historicamente determinado por uma comunidade cultural capaz de instruir, replicar e modelar uma miríade de classes de comportamentos complexos ^(^ ^14^ ^,^ ^25^ ^)^ . 

 Destaca-se a lacuna na produção de evidências empíricas sobre o trabalho de enfermagem em saúde mental na Atenção Primária à Saúde, em especial nas situações de crise, com fundamentação crítica e com uso de instrumentos validados, como o *Theorical Domains Framework* (TDF). Isso ocorre principalmente no Brasil, onde a implementação do instrumento na temática ainda é pequena, necessitando urgentemente de identificação de barreiras, tendo em conta o aumento expressivo dos problemas de saúde mental mundialmente ^(^ ^1^ ^,^ ^26^ ^)^ . 

Assim, o estudo teve como objetivo analisar o trabalho do enfermeiro na APS diante de situações de crise de saúde mental, partindo dos relatos verbais desses profissionais.

## Método

### Tipo do estudo e desenho teórico-metodológico

Trata-se de um estudo descritivo-exploratório, com abordagem qualitativa, utilização da AC e do MHD como referenciais teóricos-interpretativos, e do TDF, como metodológico-analítico.

 O TDF é um instrumento composto por um conjunto de 14 domínios e seus respectivos constructos e visa auxiliar na identificação das influências e barreiras (“cognitivas”, ambientais e sociais) que determinam um comportamento. Ele sugere as seguintes etapas: 1) seleção e especificação do comportamento alvo — o trabalho de assistência do enfermeiro; 2) seleção do desenho do estudo; 3) estratégias de seleção de participantes; 4) desenvolvimento de roteiro de entrevista; 5) coleta e análise de dados. Para revisão e escrita do relatório, foi utilizado o *Consolidated Criteria for Reporting Qualitative Research* (COREQ) ^(^ ^26^ ^-^ ^27^ ^)^ . 

 Para a interpretação e síntese, foram utilizados conceitos-chave dos referenciais teóricos interpretativos. Quanto à AC, foram utilizados os conceitos de história de aprendizagem, determinantes históricos do comportamento (filogenia, ontogenia e cultura), eventos reforçadores positivos e negativos e padrões de reforçamento. Com relação ao MHD, foram utilizados os conceitos de trabalho (concreto e abstrato), valor (uso e de troca), capital, mercadoria, alienação, conflito de classes, relações de produção, exploração, ideologia, precarização, neoliberalismo, relação centro/periferia e fetiche ^(^ ^14^ ^,^ ^25^ ^)^ . 

### Cenário

O estudo foi realizado em seis Unidades Básicas de Saúde (UBS) e seis Unidades de Saúde da Família (USF), de uma capital da Região Centro-Oeste brasileira. A rede de atenção à saúde do município continha 61 USF e 11 UBS, distribuídas em sete regiões de saúde.

### Período

A coleta de dados foi realizada entre outubro de 2022 e fevereiro de 2023.

### Critérios de seleção

 Para a seleção e a especificação do comportamento, foi utilizada a estratégia *Who* (enfermeiros), *When* (contato do usuário em situação de crise de saúde mental), *Where* (Atenção Primária à Saúde) e *How* (práticas e ações associadas ao trabalho de enfermeiro) ^(^ ^26^ ^)^ . 

A população do estudo foi definida como: enfermeiros com vínculo empregatício e atuação nas USF e UBS. Para a seleção dos participantes, foram estipulados os seguintes critérios de inclusão: ser enfermeiro, estar lotado e ter atuação no cargo de enfermeiro na USF ou na UBS por, no mínimo, seis meses.

O critério do tempo de atuação foi escolhido arbitrariamente pelos autores, por meio de discussão em grupo de trabalho considerando o tempo necessário para que o profissional se habitue ao contexto de trabalho e conheça sua comunidade. Foram excluídos os profissionais que estivessem afastados e/ou em período de férias.

### Participantes

 Participou do estudo um enfermeiro de cada unidade de saúde. Cada USF e UBS apresentava, em média, dois profissionais enfermeiros. Os pesquisadores optaram pela participação de um profissional por unidade para as entrevistas terem maior diversidade de locais/unidades, sem atingir o critério de saturação de 10 entrevistas seguidas das três adicionais ou até não haver presença de novos temas ^(28).^

Como estratégia para a seleção dos participantes, as unidades foram divididas em sete grupos (G1 a G7) e se procedeu à numeração das UBS e das USF de cada região de saúde (G1:UBS1; G2:UBS2; G1:USF1; G2:USF1 e assim por diante).

Em seguida, cada grupo de unidades foi inserido em uma ferramenta de sorteio online, para seleção de uma UBS e uma USF de cada região de saúde. A unidade sorteada era excluída se houvesse recusa de todos os profissionais da unidade. Foram sorteadas, inicialmente, quatorze unidades de saúde, sendo sete UBS e sete USF. Dessas, apenas uma era conhecida pelo autor principal da pesquisa. Nesse caso, a entrevista foi realizada por outro pesquisador.

Um total de treze enfermeiros aceitou participar do estudo, entretanto, um deles foi excluído, por dificuldades em receber, após três tentativas, os pesquisadores no dia/local agendado para a realização da entrevista. Assim, um total de doze profissionais, distribuídos nas sete regiões de saúde da cidade, participaram do estudo.

### Coleta de dados

A coleta de dados ocorreu por meio de uma única entrevista semiestruturada, individual, presencial, gravada em mídia de áudio, com o uso de gravador digital, sem a gravação da imagem, com duração média de quarenta e cinco minutos.

Para isso, utilizou-se roteiro semiestruturado com questões sobre o conhecimento dos profissionais, estratégias e habilidades utilizadas, intenções e metas, efetividade do manejo, papel do profissional, apoios sociais e profissionais, barreiras, facilitadores e sentimentos. Também foi utilizado um instrumento de caracterização dos participantes com idade, estado civil, tempo da graduação, cor/etnia, carga horária, tempo no cargo e renda.

A coleta de dados foi realizada por dois pesquisadores, sendo uma aluna de graduação e um enfermeiro (cursando o mestrado), ambos treinados previamente.

### Procedimentos para a coleta de dados

Para o início da coleta de dados, foi realizado o contato telefônico com o responsável pela unidade, acordando um horário para a apresentação da pesquisa, de maneira que não comprometesse a rotina e os atendimentos da população.

Nessa visita, os pesquisadores responsáveis pela coleta de dados realizaram, para todos os enfermeiros da unidade, a apresentação da proposta de pesquisa e procedimentos de coleta de dados.

Após esse primeiro contato com os enfermeiros, foram obtidos três tipos de respostas: 1) o não aceite em participar; 2) o aceite com agendamento da entrevista; 3) o aceite com realização da entrevista naquele momento. No primeiro caso, a unidade foi excluída da lista e um novo sorteio foi realizado no grupo da unidade visitada. Dentre as razões citadas pelos profissionais para recusar participar, estão a carga de trabalho e a indisponibilidade de colegas para substituir o profissional durante a entrevista.

Na segunda situação, a entrevista foi agendada em um dia/local escolhido pelo participante, sendo ele informado de que poderia cancelar ou reagendar a qualquer momento. Quando alguma situação do participante impedia a realização da entrevista, foi realizado o agendamento de uma nova data, com o prazo de até sete dias da data inicial.

Nas unidades em que mais de um profissional por unidade manifestou interesse em participar, os pesquisadores realizaram nova orientação quanto à metodologia e o profissional foi inserida na lista reserva para utilização em situações de impossibilidade de realização com o profissional inicialmente selecionado. Na terceira situação, a entrevista foi realizada no momento do aceite.

### Tratamento e análise dos dados

Todos os áudios das entrevistas foram transcritos na íntegra e as identificações dos participantes, de pessoas e instituições mencionadas foram substituídas por códigos de domínio apenas dos pesquisadores. Para cada entrevista realizada, foi atribuído um código que descrevia a ordem da entrevista realizada e o grupo ao qual ela pertencia (ex: G1:E1, G2:E2, G3:E3, e assim por diante).

 O instrumento TDF ( Figura 1 ) forneceu uma estrutura dos fatores determinantes (os domínios e constructos) para o trabalho do enfermeiro diante de situações de crise em saúde mental, ou seja, facilitando a identificação dos determinantes que estabelecem, mantêm e modificam o comportamento ^(^ ^26^ ^)^ . 

 Para o processo de análise foi utilizado o método dedutivo, envolvendo cinco fases: 1) leitura e exploração do material; 2) codificação e emparelhamento do conteúdo das falas nos constructos e domínios que melhor refletisse o conteúdo transcrito; 3) distribuição absoluta e proporcional do número de falas; 4) elaboração dos temas considerando as intersecções das falas e os referenciais teóricos interpretativos; 4) construção de um mapa ilustrativo das relações entre os temas/domínios ^(^ ^26^ ^,^ ^29^ ^)^ . 

**Figura 1:** - *Theorical Domains Framework* : seus domínios, definição e constructos

**Domínio**	**Definição**	**Constructo**
1.Conhecimento	Uma consciência da existência de algo.	-Conhecimento (incluindo conhecimento da condição/fundamentação científica) -Conhecimento processual -Conhecimento do contexto da tarefa
2.Capacidade	Uma habilidade ou proficiência adquirida através da prática.	-Desenvolvimento de capacidades -Competência -Habilidade -Capacidades interpessoais -Prática -Avaliação de capacidades
3.Identidade e papel profissional/social	Um conjunto de comportamentos coerentes e a exibição de qualidades pessoais de um indivíduo num ambiente social ou de trabalho.	-Identidade profissional -Papel profissional -Identidade social -Identidade -Limites profissionais -Confiança profissional -Identidade de grupo -Liderança -Comprometimento organizacional
4.Crenças sobre capacidades	Aceitação da verdade, realidade ou validade sobre uma habilidade, talento ou facilidade que uma pessoa pode usar de forma construtiva.	-Autoconfiança -Competência percebida -Autoeficácia -Controle comportamental percebido -Crenças -Autoestima -Empoderamento -Confiança profissional
5.Otimismo	A confiança de que as coisas acontecerão da melhor forma ou de que os objetivos desejados serão alcançados.	-Otimismo -Pessimismo -Otimismo irrealista -Identidade
6.Crenças sobre consequências	Aceitação da verdade, realidade ou validade dos resultados de um comportamento em uma determinada situação.	-Crenças -Expectativas de resultados -Características das expectativas de resultados -Arrependimento antecipado -Consequentes
7.Reforço	Aumentar a probabilidade de uma resposta pelo estabelecimento de uma relação de dependência ou contingência entre a resposta e um determinado estímulo.	-Recompensas (proximais/distais, com valor/sem valor, prováveis/improváveis) -Incentivos -Punição -Consequentes -Reforço -Contingências -Sanções
8.Intenções	Uma decisão consciente de desempenhar um comportamento ou uma determinação para agir de certa maneira.	-Estabilidade de intenções -Estágios de modelo de mudança -Modelo transteórico e estágio de mudança
9.Metas	Representações mentais de resultados ou estados finais que um indivíduo deseja alcançar.	-Metas (distais/proximais) -Prioridade das metas -Meta/Definição de metas -Metas (autônomas/controladas) -Plano de ação -Intenção de implementação
10.Processos de memória, atenção e decisão	Habilidade para reter informações, focar seletivamente em aspectos do ambiente e escolher entre duas ou mais alternativas.	-Memória -Atenção -Controle da atenção -Tomada de decisão -Sobrecarga cognitiva/cansaço
11.Contexto ambiental e recursos	Qualquer circunstância da situação ou do ambiente de uma pessoa que desencoraja ou encoraja o desenvolvimento de capacidades e habilidades independência, competência social e comportamento adaptativo.	-Estressores ambientais -Recursos materiais -Cultura/clima organizacional -Eventos salientes/incidentes críticos -Interação pessoa x ambiente -Barreiras e facilitadores
12.Influências sociais	Aqueles processos interpessoais que podem fazer com que os indivíduos mudem seus pensamentos, sentimentos ou comportamento.	-Pressão social -Normas sociais -Conformidade de grupo -Comparações sociais -Normas de grupo -Suporte social -Poder -Conflito entre grupos -Alienação -Identidade de grupo -Modelagem
13.Emoção	Um padrão de reação complexo, envolvendo elementos experienciais, comportamentais e fisiológicos, pelo qual o indivíduo tenta lidar com um assunto ou evento pessoalmente significativo.	-Medo -Ansiedade -Afeto -Estresse -Depressão -Afeto positivo/negativo -Burnout
14.Regulação comportamental	Qualquer coisa destinada a gerenciar ou mudar ações objetivamente observadas, ou medidas.	-Automonitoramento -Quebrando hábitos -Plano de ação

Fonte: Retirado do guia de utilização do *Theorical Domains Framework* ^(^ ^26^ ^)^

 Esse processo foi realizado de maneira independente, por dois pesquisadores treinados no uso do instrumento e, posteriormente, a análise foi comparada e discutida até o consenso. Quando o consenso não pode ser alcançado, a fala/trecho da entrevista foi alocada em todos os domínios elencados pelos pesquisadores ^(^ ^26^ ^)^ . 

 Toda essa análise foi guiada pela construção de quadros contendo as falas/trechos dos participantes. A interpretação das falas foi realizada tendo como base as definições dos constructos descritas no TDF e contraposição aos referenciais teóricos da AC e do MHD. Os dados de caracterização da amostra e a distribuição quantitativa (absoluta e proporcional) foram organizados e analisados em um documento de *Excel* ( *Microsoft* ). 

### Aspectos éticos

O estudo foi aprovado pelo Comitê de Ética em Pesquisa Envolvendo Seres Humanos, conforme CAAE: 59722622.9.0000.0021.

## Resultados

Participaram do estudo doze profissionais, sete eram do sexo masculino (58,3%), com média etária de 33 anos (máximo 47, mediana de 31,5 e mínimo 24), a maioria solteiros (58,3%), oito (66,7%) identificaram-se como como pardos, dois (16,7%) como brancos e um como negro (8,3%). Quanto ao tempo no cargo, houve variação entre um e dez anos, com uma média de três anos e meio. A renda média familiar dos profissionais foi de R$ 8.550 reais.

 Com a análise, foram identificados constructos dos 14 domínios, com a seguinte distribuição ( Tabela 1 ). 

 Com a análise e interpretação, os dados foram agrupados em dois temas, sendo eles “O trabalho do enfermeiro: determinantes materiais e sociais (TEMA-01)” e “Condições subjetivas do enfermeiro diante de situações de crise de saúde mental (TEMA-02)” (ver Figura 2 ). 

**Tabela 1:** - Distribuição absoluta e proporcional (por domínio e em relação ao total) dos constructos e domínios. Campo Grande, MS, Brasil, 2022-2023

**Número de eventos**	Proporção por domínio (% ^*^ )	**Constructo**	**Domínio**
61	14,48	Conhecimento (incluindo conhecimento da condição/fundamentação científica)	Conhecimento
74		Conhecimento processual	
97		Conhecimento do contexto da tarefa	
44	17,01	Recursos/recursos materiais	Contexto Ambiental e recursos
3		Estressores ambientais	
15		Cultura/clima organizacional	
96		Barreiras e facilitadores	
10		Eventos salientes/incidentes críticos	
98		Interação pessoa X ambiente	
9	14,61	Capacidade	Capacidades
15		Desenvolvimento de capacidade	
59		Competência	
20		Capacidades interpessoais	
31		Prática	
55		Avaliação de capacidades	
42		Habilidades	
49	9,55	Identidade Profissional	Identidade e Papel profissional/social
65		Papel profissional	
3		Liderança	
34		Identidade de grupo	
10	8,16	Autoconfiança	Crenças sobre capacidades
5		Competência percebida	
6		Autoeficácia	
43		Crenças	
3		Empoderamento	
44		Controle Comportamental Percebido	
18		Confiança profissional	
23	7,21	Crenças	Crenças sobre consequências
25		Expectativa de Resultados	
15		Características das expectativas de resultados	
9		Arrependimento antecipado	
42		Consequentes	
1	1,20	Identidade	Otimismo
7		Otimismo	
9		Pessimismo	
2		Otimismo irrealista	
17	14,17	Recompensa (proximal/distal, válido ou não válido, provável ou improvável)	Reforço
1		Incentivos	
49		Punição	
43		Consequentes	
21		Reforço	
92		Contingências	
1		Sanções	
8	0,51	Estabilidade de intenções	Intenções
17	3,92	Metas (distais/proximais)	Metas
5		Metas (autônomas/controladas)	
13		Meta/Definição de meta	
13		Prioridade de metas	
12		Intenções de implementação	
2		Plano de ação	
4	2,97	Pressão social	Influências sociais
1		Comparações sociais	
1		Conformidade grupal	
1		Normas sociais	
37		Suporte social	
2		Conflito entre grupos	
1		Poder	
2	3,35	Medo	Emoções
1		Ansiedade	
27		Afeto	
0		Estresse	
17		Afeto Positivo/Negativo	
6		Esgotamento	
13	0,82	Automonitoramento	Regulação comportamental
1		Quebrando hábitos	
18		Plano de ação	
1	2,02	Memória	Memória, atenção e processo decisório
11		Controle de atenção	
8		Atenção	
11		Tomada de decisão	
1		Sobrecarga cognitiva/cansaço	

^*^ Valor em porcentagem da distribuição proporcional (relativa) de cada domínio

**Figura 2:** - Mapa dos temas e as intersecções entre os domínios do TDF*

* *Theorical Domains Framework*

Notas: Linha verde: relação única com Tema 01; Linha azul: Relação única com Tema 02; Linha vermelha: relação de entre os dois temas; Linha tracejada: relação entre constructos; elaboração própria, baseada na literatura ^(^ ^29^ ^)^

### O trabalho do enfermeiro: determinantes materiais e sociais

As atividades relacionadas ao trabalho de assistência do enfermeiro foram relatadas nos domínios de capacidade e de conhecimento processual, emergindo práticas como: encaminhamento dos usuários para o Serviço de Urgência e Emergência (SUE) ou especializados como o CAPS, Núcleos de Apoio à Saúde da Família (NASF); internação; classificação de risco; contenção medicamentosa e/ou mecânica; acolhimento, visita domiciliar em casos excepcionais e busca ativa.

*(...) duas alternativas, uma é acionar o atendimento de urgência e emergência para poder conter o próprio, para ele não se automutilar [...].* (E1) 

*(...) entrou aqui na unidade, ele passa por uma escuta, se é óbvio que ele está num momento de muita crise [...] é tratado como uma urgência mesmo, a maior classificação daqui. [...] ele ser* á *detectado e ser* á *mandado para o consultório de enfermagem para ele fazer uma classificação de risco [...] encaminhamos os pacientes via sistema de vagas [...]* (E08) 

*(...) encaminhamos para o CAPS, porque ele vai ser avaliado lá, já passo para o atendimento com psicólogo, às vezes com psiquiatra e se for o caso de internação.* (E10) 

*Acolhimento. As pessoas vêm precisando de atenção e afetividade, entendendo que alguém esteja olhando por ela, sem julgar. Então, acho que esse é o ponto-chave da Atenção Primária [...] acolher o usuário dentro da sua individualidade.* (E09) 

Descreve-se o enfermeiro (identidade e papel profissional/social) como o profissional com maior contato com os usuários, responsável pelo acolhimento, apoio e diálogo e pela classificação/organização do atendimento. Ademais, foi citada a responsabilidade pela articulação entre equipe, família e com componentes da RAPS.

*A enfermagem é o primeiro contato quando eu estou falando com o paciente [...] mas a gente tem mais, vamos dizer assim, familiaridade até com as famílias [...] E de dar apoio emocional também, a gente tem essa função de tratar o emocional do paciente, ofertar o apoio.* (E02) 

*Se o paciente não está estável tentamos estabiliz* á-lo *aqui, passa pela consulta de enfermagem, faz-se a classificação e aí é encaminhado para o médico que estabiliza o quadro com alguma medicação [...]* . (E08) 

*Olha, o enfermeiro eu o vejo como uma pessoa que vai organizar o atendimento, direcionar bem o atendimento* [...]. (E10) 

*Levantamos a demanda e é o enfermeiro quem articula esse processo, inclusive com a família, sendo que nós* é *que estaremos na casa para orientar e conseguir trazer a equipe multidisciplinar para discutir os casos* . (E03) 

*Temos o nosso papel também, como posso te dizer, tentar trazer a resolutividade também para esses casos. [...] ...para esse quadro que o paciente vem apresentando.* (E05) 

As ações e práticas realizadas na APS, segundo os profissionais, têm consequências na adesão ao tratamento (melhoria/piora); dificuldades no seguimento e na identificação dos casos; quebra de vínculo e recorrência de crises.

*E a outra coisa é a adesão. Adesão ao tratamento. Então, o paciente acha que está bom e aí não adere mais ao tratamento e aí vem de novo a crise* . [...]. (E03) 

*Então, quando a gente utiliza esse método de assistência que é acolher, escutar, acabamos tendo uma adesão muito grande dos pacientes [...]. Quando ele consegue perceber, ter a percepção do processo saúde e doença, isso facilita a adesão ao tratamento [...] auxilia na mudança de estilo de vida [...].* (E05) 

*(...) então, assim, o paciente não cria o vínculo com seu médico da equipe, não cria vínculo com o enfermeiro que representa a equipe inteira. [...] então, como que o paciente criará vínculo se toda vez que ele vier aqui é um médico diferente? [...].* (E08) 

Em relação ao contexto ambiental e de recursos, os enfermeiros relatam: número insuficiente de vagas, dificuldades de acesso aos serviços (tempo de espera prolongado, por exemplo), sucateamento das estruturas e de espaços adequados de atendimento, recursos humanos insuficientes e alta rotatividade. Esse contexto foi relacionado a consequências como sobrecarga profissional e diminuição do número de profissionais para atendimento ao usuário.

 (...) *um desgaste muito grande, no profissional de quarenta horas, tem muito sofrimento com isso dentro desse processo, com toda essa carga [...].* (E09) 

*(...) E a minha preocupação também se refere às vezes* à *diminuição da oferta pequena de vagas para tratamento oportuno desses pacientes.* (E02) 

 (...) *aí a pessoa entra numa fila, demora 5 a 6 meses para conseguir um atendimento* [...]. (E01) 

*(...) a sobrecarga do Sistema Único de Saúde (SUS) mesmo, sobrecarga do próprio sistema e a falta de profissionais, um sucateamento do SUS mesmo que está acontecendo no país* [...]. (E04) 

 Às vezes *, a estrutura física não tem sala o suficiente, é uma sala para quatro enfermeiros, então muitas vezes não tem para onde levar esse paciente para conversar com melhor.* (E12) 

Sobre as influências sociais (suporte social, pressão social, conflito entre grupos) foi descrita a presença de apoio para direcionamento aos serviços especializados (CAPS e NASF) e aos Serviços de Urgência e Emergência (SUE), mas com comprometimento na comunicação e contrarreferência. Interferências da equipe médica na autonomia do enfermeiro também foram citadas.

*(...) não conversa, não tem contrarreferência, não tem aquela certa autonomia de que mandarei o paciente e terei acesso a tudo que está sendo feito [...]. Então é um looping infinito que temos que ficar indo atrás, não tem isso [...].* (E08) 

*(...) E posso ligar para o bombeiro, posso ligar para o Serviço de Atendimento Móvel de Urgência (SAMU) também [...]. (E02)*

*(...) porque eu não tenho acesso, ou minha categoria não permite, ou o que posso fazer, às vezes,* é desmanchado por outro profissional, pela questão médica [...]. (E12) 

As atividades de educação/discussão mensais também foram relatadas como uma forma de suporte/apoio social.

*Todo mês temos reuniões mensais que acontecem lá no Centro Especializado Municipal (CEM) com a equipe de saúde mental, onde são abordados vários temas referentes ao fluxo de pacientes e demandas da regulação. Fazemos discussão de caso, levamos casos reais que acontecem nas unidades para discutir para ver o que poderíamos ter realizado, elencamos as potencialidades e as fragilidades desse atendimento.* (E05) 

### Condições subjetivas do enfermeiro diante situações de crise de saúde mental

Os enfermeiros relataram insegurança/falta de capacidade técnica, além de situações onde foi expresso um estigma direcionado aos usuários.

*(...) a falta de capacidade técnica mesmo [...] tento o melhor enquanto enfermeiro, tudo baseado em tudo que apreendi, mas, eu sou uma especialista nisso? Não! Faço parte de um grupo que sempre tem educação permanente sobre isso para saber manejar os casos? Não!* (E08) 

 (...) É *, tem alguns preconceitos de caráter religioso, de caráter social, caráter econômico [...] acho que um dos fatores é a discriminação ainda com paciente mental. Muitas vezes falam serem possessões demoníacas [...] falam que o paciente é “vagabundo”.* (E12) 

A crise de saúde mental (conhecimento da condição) foi associada à noção de urgência/emergência psiquiátrica, e, por vezes, o termo “surto” foi utilizado. Ademais, os participantes, ao especificarem os tipos de crise, citaram sinais/sintomas de transtornos mentais.

*A crise é o surto psiquiátrico, eu considero ser uma situação em que o indivíduo está fora de si, isso falando em termos de urgência [.…].* (E02) 

*Pode ser desde uma crise de ansiedade, uma crise de pânico, pacientes em surto por conta de algum tipo de transtorno, transtorno bipolar, pacientes esquizofrênicos em algum tipo de surto [...].* (E06) 

Como exceção, destacam-se dois relatos que descreveram uma compreensão da crise de forma mais alinhada ao modelo psicossocial.

*Então, eu entendo a crise em saúde mental como um processo [...], mas algo inesperado que o paciente por si só não tem condições de estar lidando com aquele momento desafiador ou ainda algum acontecimento externo [...].* (E04) 

 (...) *Acho que é todo aquele agravo que a pessoa não consegue mais ter um bom discernimento, psíquico e mental, que acaba interferindo nas atividades diárias, tanto em relação familiar como na relação de amigos e de trabalho. Acaba que a pessoa acaba tendo comprometimento no seu processo de vínculos sociais... [...]* (E11) 

Em relação às crenças (capacidades e consequências), os participantes relataram acreditar que reduzem os riscos, mas que seu trabalho não auxilia no tratamento dos transtornos, mas sim, apenas controla pontualmente as crises.

*(...) não que evita um problema maior, mas conseguimos minimizar os riscos de gravidade da doença ou de um surto em si, ou de tentativa de suicídio [...] uma redução de danos [...].* (E02) 

*(...) a causa do problema ela não foi solucionada, aquele evento desencadeante, às vezes, nem foi resolvido [...] eu acho que a nossa assistência hoje está fazendo o paciente ficar viciado em medicação, não está auxiliando o controle da doença em hipótese alguma.* (E06) 

Eles destacam que buscam com seu trabalho (metas): redução do sofrimento, adequação e regularização do tratamento medicamentoso, vinculação a serviços especializados e estabilização dos usuários para retornarem ao convívio em sociedade.

*Aí, a maior meta é reduzir o sofrimento psíquico do paciente, porque não adianta nada eu ter uma fila zerada, mas tenho usuário sofrendo absurdamente a dor psicológica, ela dói mais do que a dor física* (E09). 

*Na verdade, quando usamos essas estratégias, tentamos trazer os pacientes para o tratamento regular [...].* (E07) 

*Na questão da pessoa em depressão, evitar que ela chegue ao estágio de realizar um autoflagelo, automutilação, o autoextermínio [...]. ... Fazer o encaminhamento para o CAPS, o Centro de Apoio Psicossocial que temos na cidade.* (E11) 

*Tentar estabilizar para eles poderem ter um convívio na sociedade normal [...] ...intenção de conseguir ingress* á-lo *na vida social de novo [...]* (E03). 

Por fim, os enfermeiros relataram emoções de angústia, medo, receio, despreparo/incapacidade, frustração, culpa e cansaço.

*Frustração, acredito que seja o mais forte. [...]. Daí vem a frustração de não poder fazer mais do que aquilo para ajudar.* (E11) 

*Então, isso gera bastante angústia no profissional [...].* (E09) 

*Tem muito profissional que não tem capacidade, tem medo, tem receio, não sabe como agir, muitas vezes na hora da crise* [...]. (E12) 

*Mas eu não me sinto assim, eu não sinto prazer em atender esse tipo de demanda, entendeu? Não é uma coisa assim que me chama muita atenção* . (E05) 

## Discussão

Os resultados reforçam a tese de que o trabalho do enfermeiro está restrito ao âmbito “técnico” focado em diagnósticos psiquiátricos, permeado por contradições objetivas e subjetivas que interferem direta ou indiretamente nas estratégias utilizadas em seu processo de trabalho e no cuidado para indivíduos em situações de crise de saúde mental.

 Desse modo, mesmo com ferramentas e tecnologias para o enfermeiro executar um trabalho que promova uma vivência positiva da crise, as condições e recursos disponíveis acabam por estimular uma execução protocolar e exaustiva, visto a sobrecarga do profissional, de etapas fixas de assistência; classificação de risco, identificação de sinais/sintomas e direcionamento dos usuários a serviços especializados; que, direta ou indiretamente, promovem o fenômeno da medicalização e patologização dos problemas de saúde mental ^(^ ^12^ ^,^ ^16^ ^,^ ^30^ ^)^ . 

 Além disso, as condições de trabalho ampliam as dificuldades na organização de estratégias de educação permanente, essenciais para o desenvolvimento de habilidades adequadas para se atender as condições de saúde; situação identificada tanto neste trabalho como também visto na literatura nacional e internacional ^(^ ^16^ ^,^ ^30^ ^)^ . 

 Nesse sentido, pode-se supor que persista na APS uma perspectiva manicomial, sustentado por um projeto neoliberal de asfixia político-econômica, pautado pela austeridade, que em um país como o Brasil, se configura pela diminuição gradual do estado e na sua capacidade de implementar políticas públicas de saúde ^(^ ^5^ ^,^ ^21^ ^-^ ^24^ ^)^ . 

 Para o MHD, esse projeto é essencial para a transformação da saúde mental e da crise em mercadoria. Para isso, a experiência de sofrimento humana precisa ser “simplificada” e colocada em critérios diagnósticos pré-estabelecidos, procedimento que facilita o direcionamento de demandas para a indústria farmacêutica, redes de saúde privadas e/ou religiosas; as comunidades terapêuticas ^(^ ^25^ ^,^ ^31^ ^-^ ^34^ ^)^ . 

 Ademais, é necessário ainda que os enfermeiros da APS, juntamente com o usuário e seus colegas, convivam com um ambiente de precarização, demarcado pela sobrecarga, baixa resolutividade, oferta limitada de serviços, alta rotatividade, dificuldade de construção de vínculos, instabilidade dos vínculos de trabalho e o incentivo à flexibilização/terceirização ^(^ ^21^ ^-^ ^23^ ^,^ ^30^ ^-^ ^32^ ^)^ . 

 Desse modo o trabalho do enfermeiro, essencialmente concreto e dotado de valor de uso intrínseco, acaba por torna-se alienado de sua função social; a produção de saúde pelo cuidado, e se constitui somente enquanto força de trabalho que executa atividades padronizadas, integrando a cadeia de valorização do capital (valor que gera valor) indiretamente, agilizando a circulação e a troca de mercadorias ^(^ ^25^ ^,^ ^33^ ^)^ . 

 Ademais, como visto no estudo, por vezes a compreensão da saúde mental se torna indistinguível dos diagnósticos psiquiátricos, atuando apenas como seu negativo (“não estar com um transtorno”), e a crise é vista como a situação aguda grave de um quadro de transtorno mental; fenômeno também presente na literatura ^(^ ^30^ ^-^ ^33^ ^)^ . 

 Trata-se da sociabilização de um *fetiche* técnico, ou seja, o subjetivo adquire um caráter objetivo, sendo simplificado, facilmente identificado, categorizado e controlado; o sofrimento adquire uma forma mais propícia para a dinâmica do mercado. A psiquiatria, desse modo, adquire poder social e coercitivo, descontextualizando os comportamentos do ambiente (atual e antecedente) do indivíduo, das variáveis reforçadoras e aversivas e da história pessoal de aprendizagem de cada indivíduo ^(^ ^14^ ^,^ ^25^ ^,^ ^31^ ^-^ ^32^ ^)^ . 

 Ou seja, os comportamentos visíveis (públicos) e as emoções (privadas) passam a ser explicados pelos transtornos mentais e toda história de vida — violência, negligência, maus tratos, etc. — é tratada, no máximo, como “fatores de risco individuais não modificáveis”. Essa forma de “explicação” é diariamente validada e compartilhada pelos aparelhos ideológicos do capital, visto a necessidade constante de gestão dos produtos (sofrimento) das contradições das relações de exploração do trabalho. O capital lucra, portanto, com o sofrimento que ele próprio gera, e mais, individualiza os determinantes desse sofrimento, culpabilizando os indivíduos pelas mazelas sociais do modo de produção capitalista ^(^ ^14^ ^,^ ^25^ ^,^ ^31^ ^-^ ^32^ ^)^ . 

 A compreensão sobre a saúde mental e as condições precárias que os profissionais enfrentam podem explicar ainda por que ações que teoricamente seriam associadas a atenção psicossocial, como o acolhimento e vínculo, se tornam etapas de um processo “técnico”, descaracterizadas de seu caráter de humanização, critério que requer “dispêndio de tempo diferenciado em comparação às demandas convencionais” ^(^ ^11^ ^,^ ^15^ ^,^ ^18^ ^,^ ^31^ ^)^ . 

 Esse panorama, cabe reforçar, não é exclusiva dos países periféricos, como o Brasil, sendo presente em todos os países, em menor ou maior nível. Por exemplo, nos Estados Unidos da América, a inexistência de um sistema público de saúde e o crescimento das desigualdades sociais vêm atingindo desproporcionalmente a população mais vulnerável, com o crescimento alarmante da população desabrigada, encarceramento em massa e agravamento de condições de saúde ^(^ ^5^ ^)^ . 

 Na China, apesar de esforços para diminuição da iniquidade do acesso à saúde, a diminuição do número de trabalhadores da saúde mental, inclusive de enfermeiros, e as dificuldades no treinamento desses profissionais vêm trazendo desafios para a organização de um sistema comunitário de saúde mental. Situação similar pode ser encontrada em outros países da Europa e do Oriente Médio ^(^ ^6^ ^,^ ^10^ ^,^ ^35^ ^-^ ^37^ ^)^ . 

 Além disso, destaca-se que, tanto no Brasil como em outros países, os próprios profissionais de enfermagem precisam lidar com as iniquidades de marcadores sociais de gênero e raça; a maioria da força de trabalho de enfermagem é composta por mulheres, e no Brasil, por mulheres negras que enfrentam dupla/tripla jornada de trabalho ^(^ ^5^ ^-^ ^6^ ^)^ . 

 Não surpreendem as repercussões emocionais e afetivas (frustração, medo, etc.) relatadas pelos enfermeiros. Diante das possibilidades e ações limitadas, baixo apoio social, ausência de educação permanente, pouca resolutividade e das condições de vida cada vez mais precárias, é justificável o sentimento de frustração do profissional, visto que as emoções são reações ao ambiente (comportamento reflexo apreendido) ^(^ ^14^ ^)^ . 

 Nesse sentido, a persistência de um profissional em construir ou não novas estratégias, tentar novas possibilidades, buscar novas redes de comunicação, dentre outras, está relacionada a um padrão específico de reforçamento na história do profissional, ou seja, a razão e os intervalos que levaram o profissional a tentar alternativas e se obteve ou não sucesso. Por exemplo, o número de vezes que um profissional tentou construir com o NASF um projeto terapêutico singular pelo número de sucessos obtidos ^(^ ^14^ ^)^ . 

 Ademais, as repercussões emocionais/psicológicas também podem ser encontradas em outros países. Os enfermeiros vêm enfrentando situações de violência, assédio moral, *bullying* de colegas de trabalho e problemas de organização que minam a resolutividade do seu cuidado. A literatura internacional vem revelando um cenário complexo de adoecimento no trabalho que, por vezes, leva a desfechos como o suicídio ^(^ ^37^ ^)^ . 

Desse modo, torna-se problemático responsabilizar apenas o enfermeiro pelos resultados no cuidado à crise de saúde mental, visto que não há condições concretas para a construção de ações relacionadas ao modelo psicossocial.

 Nesse sentido, individualizar a problemática apenas aumentaria o número de situações capazes de reforçar emoções “negativas”; frustração/culpabilização. Adiciona-se a isso a fragilidade na estruturação sistemática de ações de educação permanente, a sobrecarga, a baixa disponibilidade de tempo e recursos inadequados que tornam improvável a aplicação sistemática das melhores estratégias de cuidado nas situações de crise de saúde mental ^(^ ^14^ ^,^ ^16^ ^,^ ^30^ ^-^ ^31^ ^)^ . 

 Não há dúvidas, e a literatura reforça esse ponto exaustivamente, da necessidade de investimento na formação e educação permanente dos enfermeiros para lidar com situações de crise. Contudo, para os investimentos serem possíveis, e para existirem condições concretas favoráveis ao desenvolvimento do cuidado psicossocial pelo enfermeiro, o estado e sua população precisam superar a política neoliberal, banindo completamente a lógica da mercadoria do campo da saúde ^(^ ^5^ ^,^ ^16^ ^,^ ^31^ ^-^ ^32^ ^,^ ^37^ ^)^ . 

Enquanto categoria com forte poder social, que convive e tem contato diário com sua comunidade, o enfermeiro pode contribuir nessa luta, por meio do fortalecimento de um protagonismo político-social com maior inserção na luta da classe trabalhadora. Deve, portanto, atuar ativamente por meio de sindicatos, conselhos, partidos de vanguarda, movimentos e iniciativas sociais, lutando por condições justas de trabalho e pela superação da pobreza, falta de moradia, saneamento, trabalho digno, dentre outros diversos problemas que assolam a sociedade capitalista.

Em relação às limitações do estudo, destaca-se que a APS é apenas um dos cenários da RAPS onde o enfermeiro pode desenvolver o cuidado de indivíduos em situações de crise de saúde mental. É vital, portanto, que novos estudos sejam conduzidos para que todo o contexto da RAPS possa ser analisado em sua completude, em um processo que envolva diversas categorias de profissionais da saúde e outros atores, inclusive os usuários.

## Conclusão

Em síntese, o estudo possibilitou analisar como está estruturado o trabalho do enfermeiro na APS diante de situações de crise de saúde mental de usuários, descrevendo suas contradições objetivas e subjetivas — uma compreensão majoritariamente manicomial e um trabalho cercado pelo fenômeno da alienação e fetichização da “técnica” que serve para a inserção indireta do trabalho concreto em saúde na cadeia de valorização do capital.

O enfermeiro tem conhecimento de que seu cuidado em situações de crise pode ser executado de outra forma, mas, diante da superexploração e precariedade de recursos, restam poucas condições para essa construção, implicando repercussões emocionais/afetivas de frustração, medo, dentre outras, nos profissionais.

Por fim, a aplicação de referenciais críticos, bem como a sistematização metodológica possibilitada pelo instrumento TDF, revela-se vital diante dessa situação, por esclarecer a necessidade de uma maior compreensão teórico-científica dos condicionantes sociais e materiais do trabalho em saúde.
